# Understanding Sensitivity in Nanoscale Sensing Devices

**DOI:** 10.1021/acsmeasuresciau.5c00023

**Published:** 2025-04-15

**Authors:** Dominik Duleba, Adria Martínez-Aviñó, Andriy Revenko, Robert P. Johnson

**Affiliations:** School of Chemistry, 8797 University College Dublin, Belfield, Dublin 4, Ireland

**Keywords:** nanopore, nanopipette, sensitivity, optimization, uncertainty, Sobol, sensor

## Abstract

In nanoscale sensors, understanding and predicting sensor sensitivity is challenging as the physical phenomena that govern the transduction mechanism are often highly nonlinear and highly coupled. The sensitivity of a sensor is related to both the magnitude of the analyte-caused signal change and the random error-caused fluctuation of the sensor’s output. The extent to which these can be controlled, by carefully designing either the geometric or operating conditions of the sensor, determines the difference in signal output between the presence and absence of the analyte, as well as the impact of random errors on the distribution of these signal outputs. Herein, we use ion-current-rectifying nanopore sensors as a simplified case study to show how geometric and operating parameters can enable sensitivity optimization. Finite element analysis is used to obtain distributions of the sensor output, and then, Sobol analysis is used to highlight the most important contributions to sensor output errors. Furthermore, the magnitude of the signal change is considered alongside the spread of the output to calculate and optimize the sensor sensitivity. We highlight that the most important parameters contributing to the output variance are geometric. We observed that as the sensor is operated at smaller pore radii and lower electrolyte concentrations, the influence of the cone angle errors increases, the influence of the pore radius errors decreases, and the output becomes broader. We also show that the highest sensitivity is expected for larger pores operated at low electrolyte concentrations, and our simulation results are validated by experimental results. Recommendations to achieve optimum sensitivity are given for a range of nanopore scenarios in which ion-rectifying nanopore sensors may be used. This work aims to provide a framework for the nanoscale community to optimize sensitivity using simulations, as the analysis highlighted herein is viable for any system that can be modeled using continuum physics.

## Graphical abstract

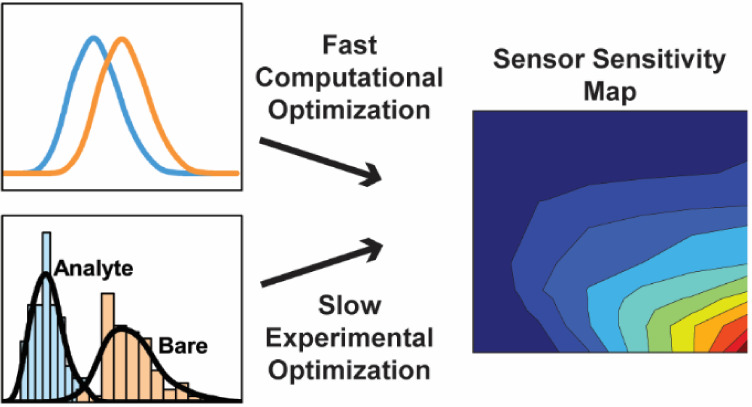

## Introduction

Nanoscale sensors and materials have at least one dimension in the nanometer range.^1^ With such reduced dimensionality, these devices and materials can exhibit physical and chemical properties not available at the macroscale.^2^ Such nanoscale sensing devices can enable ultrasensitive analysis, as well as analysis with high spatial or temporal resolution.^3,4^ The ultrasensitive analysis can be attributed to properties such as large relative surface area,^5^ fast response times,^6^ confined responsive regions,^3^ and the potential for isolation and preconcentration of analytes.^7^ Optimizing the sensitivity of these sensors with respect to both their operating conditions and geometric design is of significant importance, particularly for viable translation into commercial products. Such optimization can be (and in the sensor community, often is) approached by trial-and-error experimentation, which does not highlight the interactions of parameters. One-variable-at-a-time experimentation is also valid but requires a high number of experiments and, depending on the chosen experimental range, may only result in a local optimization.^8^ Alternatively, design of experiment (DoE) approaches can be used to simultaneously test and optimize multiple parameters using factorial design matrices;^8–10^ however, depending on the complexity and number of potentially relevant parameters, even this may require considerable experimentation. For nanoscale devices, particularly in the early stages of research at a low technology readiness level (TRL), throughput of device preparation and measurement, as well as reproducibility, is low,^11^ making such optimization work time-consuming. Depending on the method of fabrication, prototyping many designs may also be very expensive, especially when a nanofabrication cleanroom facility must be used. The analysis and optimization of sensor sensitivity via computational tools are, therefore, particularly attractive for nanoscale devices. Finite element analysis (FEA) has the ability to take a set of input parameters and correlate them to an expected output, even if the governing physical phenomena are highly coupled and nonlinear, and the geometry irregular.^12–14^ This provides a tool to investigate the influence of each input parameter on the sensor’s sensitivity with minimal experimentation. We emphasize our use of terminology at this point. In the field of statistics, formally, sensitivity refers to the impact of input uncertainties on the output’s uncertainty.^15^ However, throughout this paper and more broadly in the sensor community, sensitivity refers to the sensitivity of a sensor, which is its ability to respond to an analyte (i.e., a combination of both the magnitude of the signal change relative to the baseline and the variance of the output signal relative to that of the baseline).

A high-sensitivity sensor generally possesses two main characteristics. First, it must have a high response (signal change) to low concentrations of analytes. Second, it must display a low output variance, meaning that the device-to-device distribution of the output is narrow, allowing small analyte-caused changes to be statistically distinguished from the baseline (Figure 1). Generally, the analyte-caused signal magnitude can be maximized by optimizing individual sensor components, such as probe loading^16^ or sensor geometry,^17^ or by adjusting other operating conditions, such as the supporting electrolyte concentration.^18^ Meanwhile, the device-to-device output variance originates from the propagation of random errors associated with the input variables (geometry, solution preparation, temperature, transduction electronics, etc.). The latter can be minimized by directly addressing the source of random errors. However, for nanoscale systems, this may be difficult, as it may require improved or different fabrication methods or measurement approaches. Alternatively, the device geometry and operating conditions can be designed such that the propagation of random errors is minimized through the behavior of the physical phenomena (Figure 1). In this work, we will consider the design-based (geometry and operating conditions) minimization of random errors and maximization of the signal magnitude. Importantly, the effects of input parameters on the signal change magnitude and output variance must be considered simultaneously, as changing an input parameter may increase the sensor’s signal magnitude but, at the same time, may greatly increase the influence of input variable random errors. This would lead to excessive broadening of the output signal, resulting in an overall decrease in sensitivity despite the increased magnitude of signal change.

**Figure 1:**
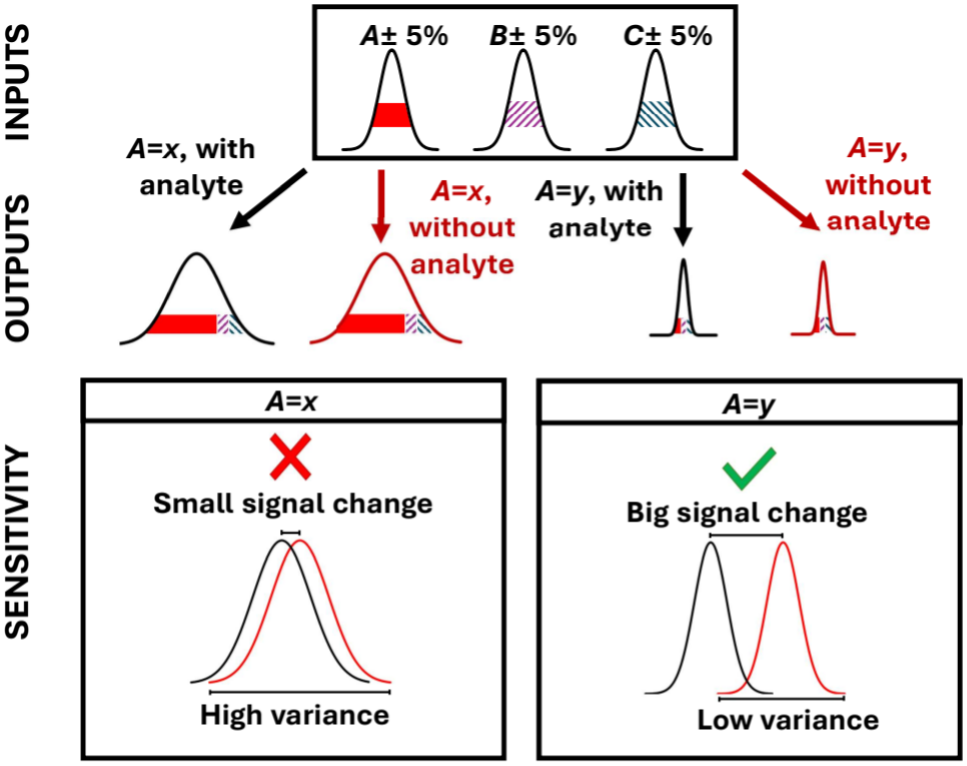
Input variables with the same relative error can propagate into an output with high variance when operated under one condition (*A* = x), or into an output with low variance when operated under another condition (*A* = y). When the distributions obtained under these two operating conditions are compared in the presence and absence of analyte, a low sensitivity sensor would exhibit a small signal change and high variance (*A* = x) and a high sensitivity sensor would exhibit a large signal change and low variance (*A* = y).

Finite element analysis can be used to investigate both the signal magnitude and the output variance, as well as how they are influenced by geometric input parameters and operating conditions, while also providing insights into the implications for overall sensitivity. The input parameter space can be sampled, taking into account the normal distribution of the input parameters arising from their random errors. Then, each sample of input parameter combinations can be solved by using FEA to obtain the expected output distribution. By having the ability to account for distributions of input variables and derive a sensor output, a range of techniques can be used to investigate the effect of each input parameter. The Morris method can be used to qualitatively identify how influential each input variable’s random errors are on the sensor’s output distribution.^19,20^ This method effectively allows for the identification of parameters that are less significant in the simulation of the output distribution, thereby reducing computational costs for further analysis. Next, Sobol analysis can be used to quantitatively identify how much of the output variance is explained by each input variable.^20,21^ Sobol analysis reveals which parameters cause the output distribution to be broad or narrow and provides insights into how parameters may be adjusted to achieve desirable output distributions. The Morris method and Sobol analysis have been previously utilized to identify the most important input parameters in flexoelectric nanostructures,^22^ batteries,^23^ nanocontact particle manipulation,^24,25^ and nanowire field-effect transistors.^26^ These methods offer a crucial fundamental understanding by pinpointing the most significant random errors. Additionally, they enable exploration of how different geometric and operating parameters influence the propagation of random errors, which is essential for optimizing the sensor’s sensitivity.

Uncertainty analysis can propagate the random errors of each input parameter toward an expected output value with its own uncertainty. This has been previously done for atomic force microscopy-based measurements, piezoelectric actuators,^27,28^ and in chemical systems such as methane-air ignition.^29^ Using a similar approach, the distributions corresponding to the presence and absence of an analyte can be calculated. The more dissimilar these two distributions are, the more likely it is that the two groups can be experimentally differentiated at low analyte concentrations. As such, this dissimilarity acts as a measure of sensor sensitivity. To investigate how changes in geometric and operating parameters affect the dissimilarity between two distributions, it is necessary to quantify said dissimilarity. Here, we use Jeffreys’ divergence, which can quantify such dissimilarity. This description of sensitivity simultaneously considers the influence of random errors associated with the input parameters (via the output variance) as well as the magnitude of sensor response (via the shifted mean values of the output distributions). Hence, the effect of each input parameter on overall sensor sensitivity can be studied.

One nanoscale sensing system that could benefit from FEA-based optimization of the geometric and operating parameters is ion-current-rectifying nanopore sensors. Ion-rectifying nanopore sensors have recently elicited great interest due to their capability for highly sensitive, label-free analyte detection.^30,31^ Nanopores with a conical geometry and pore sizes similar to the thickness of the electric double layer exhibit nonlinear current–voltage curves.^32,33^ This diode-like behavior is a result of the interactions between the ionic flux (when a potential is applied across the nanopore) and the surface charge density on the internal nanopore walls.^34–39^ Analyte-specific probes can be grafted onto the nanopore surface, resulting in a modulated net surface charge density upon analyte binding and a quantifiable change in the diode-like current–voltage curve (Figure 2).^30,31^ Most commonly, the outputs of these sensors are described by the rectification ratio, defined as I­(−V)/I­(+V). Based on changes to the rectification ratio, the analyte can be quantified; however, reproducibility is often low.^40–44^

**Figure 2:**
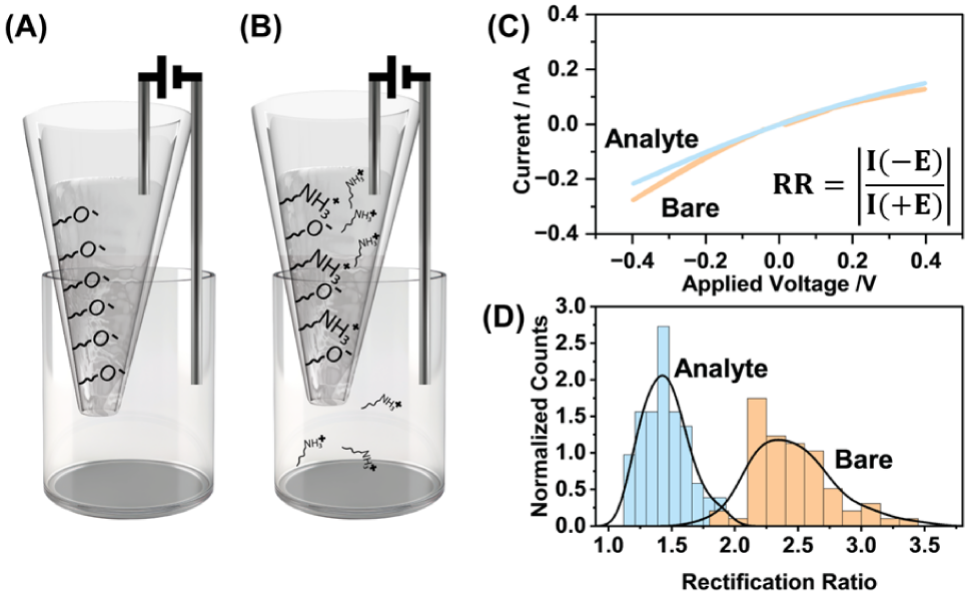
(A) Schematic of the nanopore sensor’s experimental setup in the absence of analyte with the negatively charged surface groups highlighted, (B) schematic of the setup in the presence of analyte with the immobilization of positively charged analyte on the surface highlighted, (C) experimental current–voltage curve showing that the immobilization of the analyte decreases the rectification ratio, and (D) experimental populations of rectification ratios measured in the presence and absence of the analyte.

In nanopore systems, the most easily controllable operating parameters are the supporting electrolyte concentration, the applied voltage, and the temperature, while the most easily controllable geometric parameter is the pore size. Although the strong influence of these parameters on the electrical nanopore output has been reported in a number of fundamental studies,^33,45–50^ to the best of our knowledge, neither the change of the electrical output upon analyte immobilization (signal change magnitude) nor the variance of the electrical output has been systematically studied in nanopore applications. It is expected that the controllable operating parameters will influence the sensitivity of the nanopore to the surface charge modulation of analyte binding (tuning the signal change); however, this phenomenon is not well understood. It is also anticipated that the output variance can be modified using these parameters, as the nanopore can operate in different regimes depending on the pore size and electrolyte concentrations,^45^ but this aspect is also poorly understood. Research groups, including ours, use a variety of supporting electrolyte concentrations (such as 1 mM,^51^ 10 mM,^52^ or 100 mM^53^) and applied voltages (−0.4 V,^54^ 0.5 V,^55^ and −1 V^56^), while the pore size used is often determined by other considerations, such as ease of filling, ease of fabrication, or the preference for using programs already established within the laboratory. For sensors that require high sensitivity, these parameters should be systematically selected to achieve increased sensor sensitivity or to enable faster and more reliable research-and-development experimentation. Unfortunately, experimentally deducing the optimum combination of these parameters is tedious. Device-to-device variation is high, requiring multiple repetitions to obtain statistically significant results. Furthermore, the device throughput is low as preparation of a sensor via surface modification of the pore interior with probe molecules can take multiple days.^16^ A thorough understanding of the controllable operating parameters affecting sensitivity and signal reproducibility is crucial to accelerate the technological readiness level of these promising nanopore sensors.

Herein, we use FEA of a nanopore sensor to carry out Morris and Sobol analysis, highlighting the influence of random errors on the system and how the pore size, electrolyte concentration, applied voltage, and temperature can alter the impact of random errors. We use the dissimilarity of the expected output distributions in the absence and presence of an aminosilane analyte as a measurement of sensor sensitivity, thus identifying the parameters at which sensitivity to the analyte is optimal (considering both the influence of random errors and the response magnitude of the sensor). Finally, the sensitivity analysis is experimentally verified. A flowchart of the computational pathway is provided in Figure 3.

**Figure 3:**
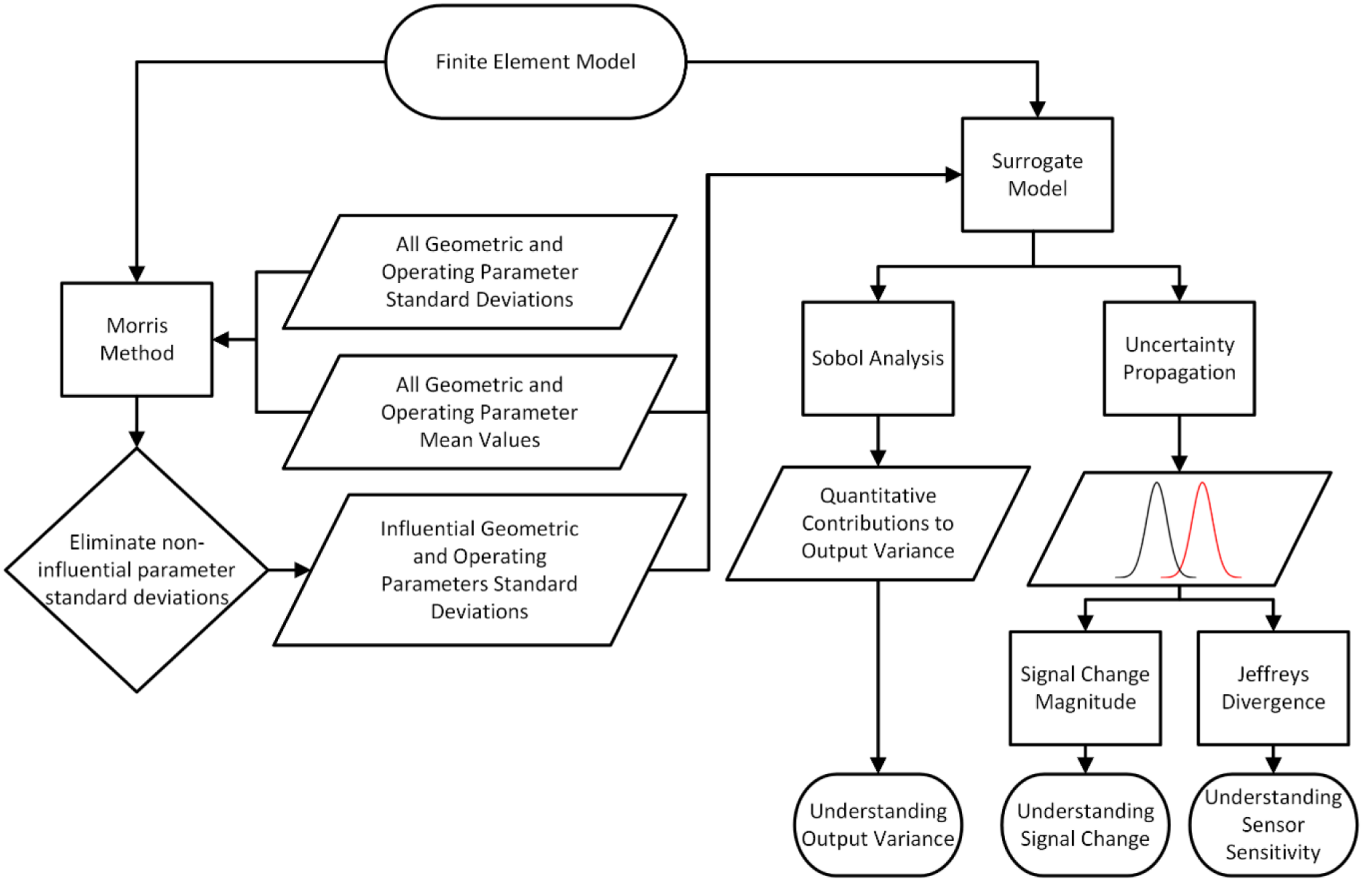
Flowchart of sensitivity analysis showing the use of the Morris method to limit the number of fluctuating geometric and operating parameters considered. A surrogate model can then be built from the desired combinations of geometric and operating parameter mean values on which Sobol analysis and uncertainty propagation will enable the understanding of the output variance, signal change, and sensor sensitivity.

To the best of our knowledge, this is the first reported systematic study of the sources of uncertainty in nanopore sensors. Furthermore, to the best of our knowledge, sensor sensitivity optimization based on FEA-aided Sobol analysis and uncertainty propagation (where the dissimilarity of the output distributions is used) has not been previously reported for nanopore sensors. The methodological demonstration of these techniques and the fundamental understanding gained are beneficial to the broader nanoscale sensor community, as this method is viable for any sensor where the underlying transduction mechanism operates on the continuum scale and can be modeled by FEA. The methodology presented herein can aid in minimizing device-to-device variation, optimizing sensitivity, and supporting sensor development by minimizing tedious experimental optimization.

## Methodology

### Finite Element Models

The commercial software COMSOL (version 6.2) is used to solve the Poisson–Nernst–Planck equations and describe the output of the nanopore sensor. Within a single model evaluation, two models, one for the positive applied potential and one for the negative applied potential, are simultaneously evaluated, and the rectification ratio is obtained. The Navier–Stokes equation is not incorporated as the model needs to be evaluated many times to construct the surrogate models that are to be sampled for Sobol analysis and uncertainty propagation. As such, the computational cost of each evaluation needs to be low. In the presented model, a single evaluation is computed in 2 min, and hence, the generation of an output distribution takes around 3 h (80 evaluations are needed for a distribution). The incorporation of the Navier–Stokes equations would extend single computation times to around 30 min and the output distribution computations to around 40 h, and the excellent agreement between experimental and simulation data that we obtain, as presented in the Results and Discussion, indicates that its inclusion is not necessary to describe the trends observed in this study. Details of the mesh, boundary conditions, physics incorporation, and geometry are available in Figures S1 and S2.

The nanopore surface charge density is described by solving a pH-responsive model that incorporates the Poisson–Nernst–Planck equations together with a Henderson–Hasselbalch-based formulation of the surface charge density, as we reported previously.^57^ The surface charge density of the bare glass is modeled as described previously;^57^ however, to describe the surface charge density after silane immobilization, the model is modified by incorporating the equilibrium constant of the surface-bound aminosilane (expression available in Supporting Information). This formulation expresses the surface charge density as a function of the acidity constants and surface group densities of the quartz’s Si–OH and the analyte’s −NH_2_ groups, as well as a function of the proton concentrations in the immediate vicinity of the surface. The local proton concentration is solved by the model and is a function of all relevant parameters, such as pore radius, cone angle, electrolyte concentration, temperature, and applied voltage. The resulting surface charge density at each studied combination of the parameters is imported into the model used herein (Table S1). The pH-responsive model provides details on the nonlinear distribution of surface charge density along the nanopore;^57^ however, for the sake of simplicity, this detail is simplified, and herein, a uniform surface charge density is applied using the average surface charge density obtained from the pH-responsive model. The treatment of temperature-dependent parameters, such as the temperature dependence of the diffusion coefficients, equilibrium constants, and the dielectric permittivity, is described in Figures S3–S5.

The building of surrogate models from finite element method evaluations, parameter sampling, Morris analysis, Sobol analysis, and uncertainty propagation is carried out using the recently released Uncertainty Quantification Module in COMSOL (version 6.2).

### Morris Screening

The screening for parameters whose random errors are influential is carried out using the Morris method. In Morris sampling, a [0,1]*^m^* hypercube is generated, where *m* is the number of input variables and the distribution of each input variable is mapped onto the hypercube (mapped onto the range of [0,1]). Data sampling starts randomly, and then, the next sample points are obtained by stepping along one dimension of the input space at a time. The direction of stepping is designed so that the trajectories formed from each starting point have the highest spread.^58^ Each sampling point is individually evaluated using the finite element model. The rectification ratios obtained for different combinations of the input variables are used to calculate the Morris mean and the Morris standard deviation.

### Sobol Analysis and Kernel Density Estimation

For the calculation of the Sobol indices, Monte Carlo analysis is carried out on a Gaussian process (GP)-based surrogate model. A GP surrogate model takes a number of FEA input evaluations, and using a mean and a covariance function, it approximates the output for new input variables without requiring new FEA evaluations (acting as a “look-up table”). An adaptive GP is used, where a sample of 30 model evaluations is used to train the GP model with a Matérn 5/2 covariance function and a quadratic mean function. An additional 50 data points are added at locations of high entropy to obtain a more accurate model.

The output distribution is also obtained via Monte Carlo analysis of the GP surrogate model. The output distribution is used to generate a kernel density estimate using a normal kernel function with a bandwidth obtained from Silverman’s rule.^59,60^

In both cases, to reduce the computational cost, only the most influential random errors (as determined from the Morris screening), i.e., the pore size, cone angle, and wall thickness, are considered.

### Approximation of Sensor Sensitivity Using Jeffrey Divergence

To model the signal change due to analyte immobilization, a change in the surface charge density is used. The critical question is what change in the surface charge density represents the immobilization of the analyte at each pore size, electrolyte concentration, applied voltage, and temperature with good validity. The simplest approach is to define some absolute change in the surface charge density, i.e., + 1 mC m^–2^. However, the magnitude of the surface charge density is different at each electrolyte concentration;^57,61^ therefore, the relative change of surface charge will be significantly different at each electrolyte concentration, potentially introducing large errors. Defining a percentage change of surface charge, i.e., + 10%, is more valid; however, this may also introduce errors arising from the complexity of physical phenomena. As the surface charge is critical, we choose to minimize our assumptions for this variable. We consider the immobilization of the analyte with a surface density of 0.01 molecules nm^–2^, which displaces a small amount of the quartz’s Si–OH groups that are present at a density of 3.8 molecules nm^–2^. Due to the displacement of some negatively charged groups and the introduction of groups that exist in a positively charged state, the surface charge density becomes more positive. Using the acidity constant value of aminosilane,^62^ along with the acidity constants associated with the glass surface, the surface charge densities before and after analyte immobilization can be calculated at each combination of pore radius, concentration, voltage, and temperature.^57^ As surface charge densities before and after analyte immobilization are calculated identically, assumptions are kept consistent, and hence, the change in surface charge density is more reliable for the analysis of sensor sensitivity. Table S1 provides a sample of the calculated surface charge densities at different electrolyte concentrations. It is evident that both the surface charge density of the unmodified surface and the percentage change upon analyte immobilization depend greatly on the supporting electrolyte concentration (4% change at 50 mM and 22% change at 0.1 mM despite immobilizing the same amount of analyte) and highlights that assuming a constant 10% change in the surface charge density could have vastly underestimated the signal change magnitude at lower electrolyte concentrations.

A separate surrogate model is trained for all combinations of the studied pore radii, concentrations, voltages, and temperatures. Each of these combinations can be studied in the absence or presence of an analyte, with a surface charge density associated with each combination. To sum up, the surface charge density is a function of the pore radius, concentration, voltage, temperature, and the absence or presence of the analyte. Jeffreys divergence is calculated for each pair (absence or presence of an analyte) of kernel density estimates obtained from the surrogate models for each combination of input variables. A MATLAB script is used for kernel density estimation and the calculation of Jeffreys divergence.

### Scanning Transmission Electron Microscopy (STEM)

STEM images were taken with a Zeiss Sigma300 FEG SEM instrument equipped with a STEM detector and operated at a 15 kV acceleration voltage. Sample images are presented in Figure S6, and the average values along with their standard deviations are shown in Table S2.

### Nanopipette Fabrication

A P-2000 laser pipette puller from Sutter was used to prepare conical quartz nanopipettes with pore radii of 17 nm (H750 F4 V40 D135 P180), 87 nm (Line 1: H700 F4 V20 D170 P0, Line 2: H680 F4 V50 D170 P200), 196 nm (H580 F3 V55 D128 P110), 232 nm (Line 1: H610 F4 V55 D150 P80, Line 2: H560 F3 V30 D135 P103), and 312 nm (H575 F3 V60 D128 P100). Capillaries were obtained from Sutter Instruments (O.D. 1.0 mm and I.D. 0.70 mm), and they were cleaned with isopropyl alcohol prior to pulling.

### Electrochemical Measurements

For electrochemical measurements, the nanopipettes were backfilled with various concentrations of KCl and dipped into a solution of the same concentration. The current–voltage curves were measured with a two-electrode (Ag/AgCl and Ag/AgCl) setup using a Biologic SP-200 potentiostat fitted with the ultralow-current (ULC) option. A scan rate of 0.05 V/s is used to scan within the −0.4 V to +0.4 V potential window. A 5 Hz filter bandwidth is used to reduce noise. The rectification ratios are extracted at −0.4 V and +0.4 V applied potentials.

At least 60 nanopores were measured for each combination of electrolyte concentration and pore radius. For the nanopores in the presence of the analyte, both the bulk and backfilled solutions contained 300 μM 3-aminopropyldimethylmethoxysilane in addition to the supporting electrolyte. The analyte is backfilled to eliminate any differences in analyte diffusion into the nanopore and to prevent asymmetric surface grafting. Sample experimental current–voltage curves are shown in Figure S7, and simulated current–voltage curves are shown in Figure S8.

## Results and Discussion

Finite element analysis allows for a set of input variables to be correlated with the output of the sensor, even if the physical phenomena that govern the transduction mechanism are highly coupled and nonlinear. However, FEA is not inherently compatible with input parameters that possess a distribution (such as a Gaussian distribution arising from random errors) as it only solves for results given individual input parameters. To address this, the distribution of each input variable (the parameter space) needs to be sampled, and each sampled combination of input variables needs to be individually solved (either directly via FEA or via a surrogate model that was trained on FEA evaluations). This produces an output with its own distribution, which is a result of the propagated random errors from the input variables. We emphasize that all of the following methods use this same principle.

### Screening Input Variables and Their Random Errors

Numerous input variables with associated random errors may contribute to the variance observed in the electrical readout of nanopore sensors. These sources may include geometric variability arising from nanopipette fabrication, such as device-to-device variability in the pore radius, internal cone angle, and wall thickness; measurement variability arising from instrumentation or from the concentrations of the prepared solutions; or variability in ambient conditions, such as temperature. Although the relative uncertainty of each parameter can be easily calculated, it cannot be easily correlated with their influence on the current–voltage outputs of the system, as these nanoconfined devices can respond in complex, nonlinear ways to variations in input. Furthermore, interactive effects between input variables may also be important to consider. Since the sensor’s sensitivity is related to the extent to which these sources of variance influence the sensor readout, it is crucial to identify which input variables are responsible for most of the variance observed. From this, improvements in the experimental or sensor design can be made, and the computational cost of further simulations can be lowered. For the initial screening of input variables, an 87 nm nanopore at 1 mM electrolyte concentration is considered, with input variables and associated random errors as shown in Table 1. This concentration was chosen as it is around the center of the experimentally relevant concentration range, and the pore radius is one that can be readily fabricated in our laboratory.

**Table 1:** Shows the Parameters and Associated Mean Values (*X̅*) and Standard Deviations (*S*) Which Are Considered for Parametric Screening via the Morris Method

Parameter	*X̅*	*S*
Pore Radius/nm	87	7
Cone Angle/deg	8.5	0.8
Wall Thickness/nm	27	7
Temperature/K	294	1
Applied Potential (+)/V	+0.4	10^–4^
Applied Potential (−)/V	–0.4	10^–4^
Concentration/mol m^–3^	1	0.02

The Morris method is widely used for the initial screening of input variables, as it is a lightweight analysis requiring few model evaluations. The Morris method provides a qualitative description of the influence of each input variable’s random errors on the sensor output distribution. This offers a qualitative understanding of which random errors are influential enough to consider in further computations. In Morris analysis, the input variable ranges are sampled for an initial set of start values, and then, one variable is changed at a time for each subsequent calculation. At each step, a different variable is adjusted while retaining the adjusted values of the other variables from previous steps. From the solutions of the sampled trajectories, a Morris mean and Morris standard deviation can be calculated. The Morris mean describes the overall effect of each parameter (where a larger number indicates a larger overall influence on the output distribution), while the Morris standard deviation describes nonlinear and interactive effects (where a larger number indicates larger effects on the input due to interactions with the other inputs).^19,58^ Figure 4 shows the Morris plot associated with the input variables and random errors listed in Table 1. The pore radius and its random errors have the most significant overall effect on the sensor’s output, as indicated by the high Morris mean. Although the cone angle and the wall thickness only have a moderate influence under these conditions, they exhibit highly interactive effects as signified by the high Morris standard deviation. These may also be important input variables if conditions are changed. In fact, running the analysis at 0.1 mM concentration shows that the cone angle and the wall thickness can have high overall influences (Figure S9). The random errors in the electrolyte concentration and temperature both have small overall and interactive effects, while the errors associated with the potentiostat voltage control are negligible. It is highlighted that the most important input variables relate to the pore geometry. To reduce the computational cost of further analysis, from hereon only the random errors associated with the pore radius, the cone angle, and the wall thickness will be considered.

**Figure 4:**
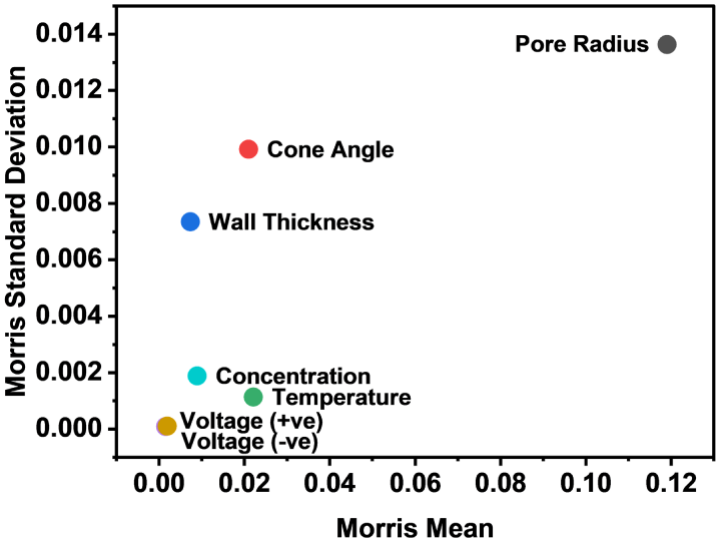
Morris plot for an 87 nm nanopipette at 1 mM supporting electrolyte concentration. Morris plots for other pore radii and supporting concentrations are given in Figure S9. The plot highlights that the most influential contributors to the output variance are geometric.

Although the only input variables whose random errors must be considered are the pore radius, the cone angle, and the wall thickness (i.e., the nanodevice geometric parameters), the mean values (not the random fluctuations) of the other input variables may also have a significant influence on the output distribution by altering how the influential input random errors propagate. Furthermore, the overall signal change of the sensor could also be affected by any of these input values. For example, although the random errors of the electrolyte concentration do not contribute to the output distribution significantly, changing the electrolyte concentration (by operating the sensor under a different salt concentration) could alter both the output distribution and the signal change magnitude. In the following sections, the practically adjustable operating parameters, namely the pore radius, the electrolyte concentration, the applied voltage, and the temperature, are investigated to understand their effects on sensor sensitivity (via the effects on the sensor output distribution and signal change magnitude). The wall thickness and cone angle are not investigated, as these parameters are not practically controlled by our laboratory. However, for completeness, the influence of the cone angle is provided in Figures S10–S11.

### Electrolyte Concentration Alters the Propagation of Random Errors, the Magnitude of Sensor Response, and Hence the Sensitivity

A more quantitative description of the influence of random errors in input parameters on the total output variance can be obtained through Sobol analysis.^20,21^ The Sobol method decomposes the total output variance into individual contributions from each input parameter. Instead of considering single trajectories from a starting point with stepwise changes to each variable, as computed by the Morris method, Sobol analysis is a variance-based method that examines the entire input parameter space distribution using Monte Carlo methods.^63^ Monte Carlo methods require more model evaluations than are feasible with FEA; therefore, FEA is used to build a surrogate model, which can then be sampled more efficiently. The Sobol index obtained from the analysis quantitatively describes the contribution of each input parameter’s variance to the output variance. Sobol indices are standardized to the total variance; thus, they indicate what percentage of the variance is explained by each parameter.

Figure 5 shows the expected sensor output distributions of 87 nm nanopipettes at various supporting electrolyte concentrations. The source of the output distribution’s variance arises from random errors in the pore size, cone angle, and wall thickness, as outlined in Table 1 (other input parameters are not considered). Figure 5A demonstrates that depending on the supporting electrolyte concentration used, the same geometric random errors can yield narrower or wider output distributions. Since Sobol analysis is quantitative, it can be used to understand the contributions that make up the output variance and reveal how each contribution changes as a function of the supporting electrolyte concentration. Figure 5B shows that the Sobol indices describing the contribution of each input parameter are functions of the electrolyte concentration. At high electrolyte concentrations, most of the output variance is explained by random errors in the pore size. As the electrolyte concentration decreases, the Sobol index of the pore radius decreases, while the Sobol index of the cone angle increases, indicating that most of the variance is now explained by random errors in the cone angle. Additionally, Figure 5A has already established that the total variance is a function of the supporting electrolyte concentration; therefore, rationalizing the Sobol indices together with the total variance would also be useful. Figure 5C shows the increasing spread of the output (visualized in terms of the total variance) as the electrolyte concentration is lowered. Initially, the increasing variance is driven by an increasing contribution from the pore radius; however, at concentrations below 1 mM, further increases in the total variance are driven by the cone angle. These results highlight that the propagation of geometric uncertainties in nanoscale sensing devices can be tuned by adjusting the operating conditions of the sensor, in this case, the supporting electrolyte concentration. A further conclusion that can be drawn is that since the cone angle is the most influential contributor to the output variance at low electrolyte concentrations, if a fabrication method allows very precise control of the cone angle (but results in high variation in the pore size), it may be worthwhile to operate the sensor at lower electrolyte concentrations to exploit this. The use of such low electrolyte concentrations could, however, introduce disadvantages such as poor signal-to-noise ratio (mid-measurement current noise rather than device-to-device fluctuation), difficulties with real sample preparation, or possible analyte incompatibilities, such as lower biomolecule stability. Conversely, in a situation where the fabrication method struggles to reproducibly control the cone angle but consistently fabricates the pore radius, using a higher supporting electrolyte concentration may be beneficial.

**Figure 5:**
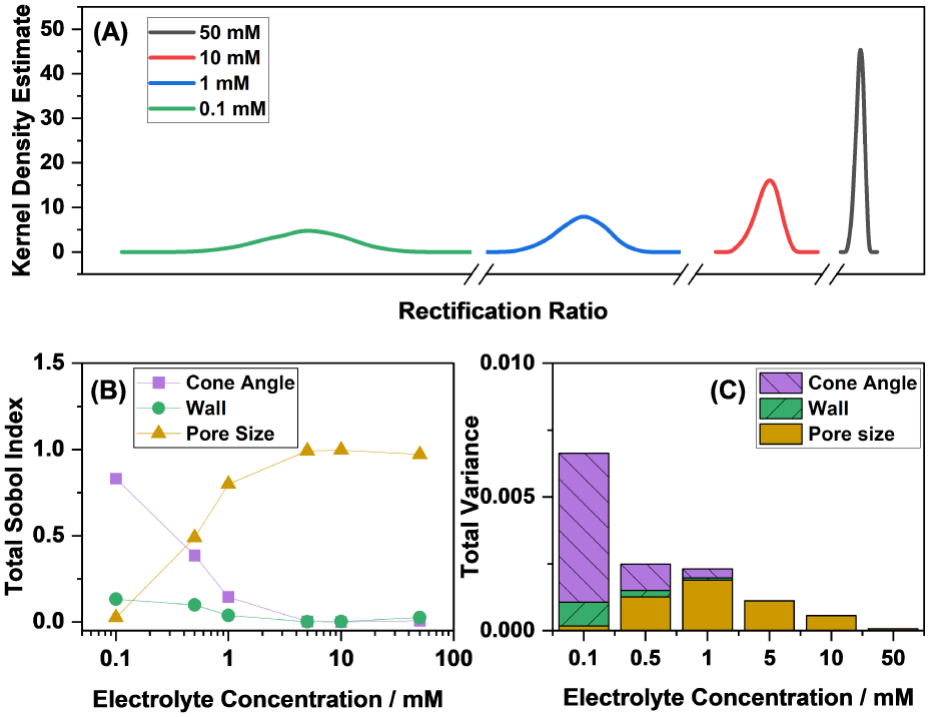
(A) Simulated output distributions of the nanopore in the absence of an analyte, (B) Sobol indices associated with each influential input variable as a function of the electrolyte concentration, and (C) total output variance as a function of the electrolyte concentration. For this simulation, a nanopore with a pore radius of 87 nm and at 8.5 degree cone angle, at 25 °C, and 0.4 V applied potential was considered.

Based on the results of Figure 5A, narrower output distributions are obtained at higher electrolyte concentrations; as such, it can be concluded that the highest sensitivity would be found here. However, this does not take into account the magnitude of the signal change, which is also a function of the supporting electrolyte concentration. To account for the signal change, the sensor’s output distributions in the absence and presence of an analyte must be computed. The presence of an analyte can be approximated by a change of surface charge density. We use the immobilization of aminosilane on glass as the model system, and the modulated surface charge density is calculated based on the immobilization of 0.01 molecules nm^–2^ of aminosilane. The surface charge densities are calculated by considering surface group protonation and deprotonation as previously developed by our group.^57^ A sample of the surface charge densities obtained before and after analyte immobilization for each condition, as a function of supporting electrolyte concentration, is provided in Table S1. Figure 6A shows a sample of the calculated output distributions in the presence and absence of the aminosilane. The signal magnitude (the difference between the means) as a function of electrolyte concentration is shown in Figure 6B. At high electrolyte concentrations, the signal magnitude is small, and then, as the concentration is decreased, the signal magnitude increases and reaches a maximum at around 0.4 mM supporting electrolyte concentration, followed by a decrease beyond this point. This behavior is related to a number of competing and interlinking phenomena. First, the rectification ratio itself exhibits a similar trend. As the electrolyte concentration is decreased, the electric double layers expand, and the ionic permselectivity increases. This leads to more extreme concentration polarization, hence an increase of the rectification ratio, as well as the signal change. At lower electrolyte concentrations, an additional ionic enrichment peak can arise close to the pore mouth,^45^ which can decrease the rectification ratio and may also affect sensitivity. Furthermore, as the concentration is varied, the location and sharpness of the electric potential drop change, effectively changing the sensing region of the nanopore.

**Figure 6:**
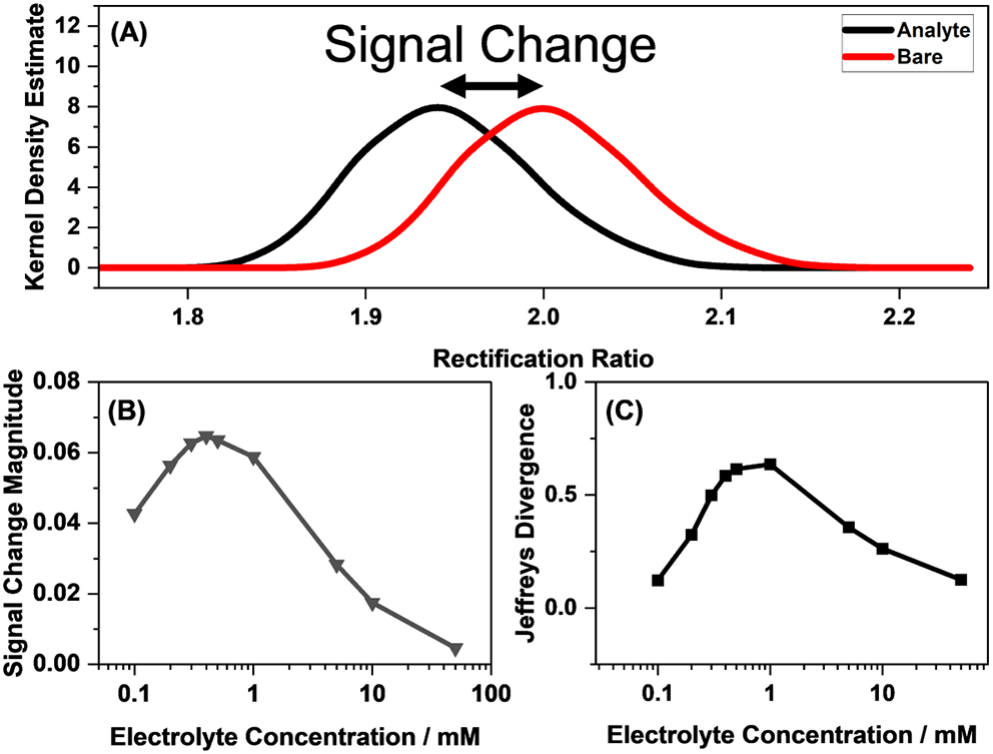
(A) Simulated distributions of the sensor output in the presence and absence of an analyte, (B) simulated signal change magnitude upon the immobilization of 0.01 molecules of nm^–2^ analyte, and (C) Jeffreys divergence as a function of the supporting electrolyte concentration. For this simulation, a nanopore with a pore radius of 87 nm and at 8.5 degree cone angle, 25 °C, and 0.4 V applied potential was considered.

The highest sensor sensitivity occurs when the pair of distributions is most distinct, meaning that measurements belonging to each distribution are most likely to be statistically differentiated from each other. For the evaluation of the dissimilarity of the two distributions, both the signal magnitudes and the variances must be considered. Kullback–Leibler divergence describes the dissimilarity of two probability distributions and hence can be used as a proxy for sensitivity:

$$D_{KL}\left(P∥Q\right)=\underset{x\in \chi }{\sum }P\left(x\right)\log\left(\frac{P\left(x\right)}{Q\left(x\right)}\right)$$

where *P* and *Q* are the probability distributions of the sensor in the presence and absence of the analyte. Herein, we use a symmetrized version of the Kullback–Leibler divergence, the Jeffreys divergence:

$$J=\frac{D_{KL}\left(P∥Q\right)+D_{KL}\left(Q∥P\right)}{2}$$

Figure 6C shows Jeffreys divergence as a function of electrolyte concentration. The nuanced usefulness of Jeffreys divergence is that it simultaneously considers both the shape of the distribution and the magnitude of the signal change. To illustrate this, it can be noted that although the highest signal change is predicted at 0.4 mM concentration, the highest sensitivity is predicted at 1 mM concentration. Furthermore, although the signal change at 0.1 mM is larger than that at 10 mM concentration, the Jeffreys divergence values are similar. This is because the variance of the output is greater at lower electrolyte concentrations, while the variance is lower at higher electrolyte concentrations. It should also be noted that if the random errors of the cone angle could be decreased, the peak sensitivity would shift to lower electrolyte concentrations (as the total variance at lower electrolyte concentrations would decrease). Decreasing the random errors of the pore radius would shift the peak sensitivity to higher concentrations.

### Pore Size Alters the Propagation of Random Errors, the Magnitude of Sensor Response, and Hence the Sensitivity

The easiest geometric parameter to control in nanopore systems is the nanopore radius. Similar to changing the supporting electrolyte concentration, the pore radius can also be used to control the influence of input variable random errors, the signal change magnitude, and the sensor sensitivity. To demonstrate this, it is necessary to assume that the geometric random errors originating from the fabrication process do not change when nanopores of different radii are fabricated. This assumption is necessary because using the actual errors associated with each nanopore size would mean that the numerically computed sensor output becomes a function of both the pore size-associated changes to the physical phenomena governing the transduction mechanism and changes to the error due to an altered quality of fabrication. The random errors in Table 1 are used, and the relative error of the 87 nm nanopore is applied to all other pore radii. The supporting electrolyte concentration is kept constant at 1 mM, and the mean values of the cone angle and wall thickness are maintained as those listed in Table 1.

Figure 7A shows that the variance of output distributions is a function of the pore radius (even though the relative errors are kept constant). At larger pore sizes, most of the variance is contributed by the error in the pore radius; however, as the pore size decreases, more of the variance is contributed by errors in the cone angle and the wall thickness (Figure 7B,C). These results highlight that changing the pore radius could be used for the optimization of sensor sensitivity. Similar conclusions can also be drawn as before. If the fabrication methodology produces highly reproducible cone angles, using a smaller pore radius may be beneficial, and vice versa.

**Figure 7:**
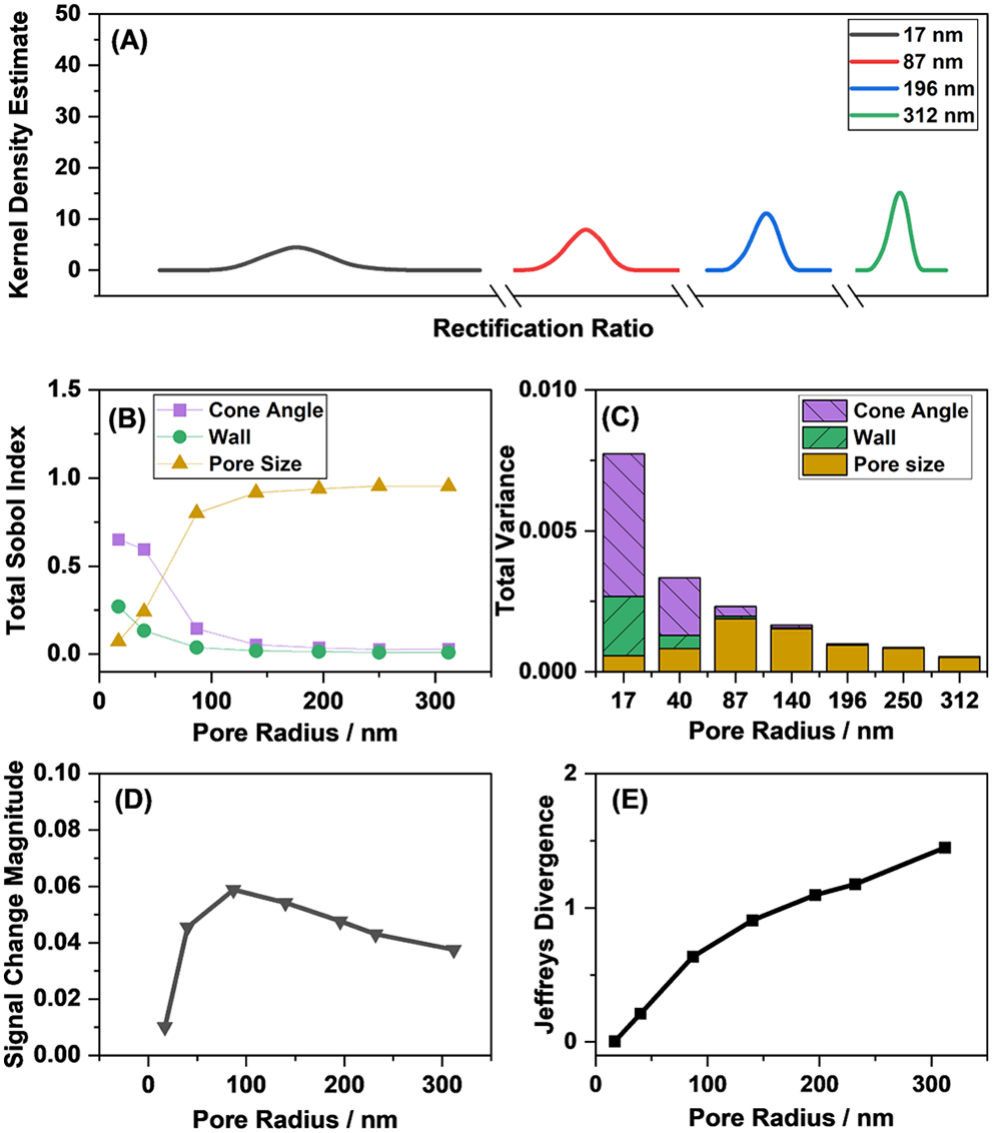
(A) Simulated output distributions of the nanopore in the absence of analyte, (B) the Sobol Indices associated with each influential input variable as a function of the pore radius, (C) total output variance as a function of the pore radius, (D) simulated signal change magnitude upon the immobilization of 0.01 molecules nm^–2^ analyte, and (E) Jeffreys divergence as a function of the pore radius. A nanopore with a 1 mM electrolyte concentration and at 8.5° cone angle, 25 °C, and 0.4 V applied potential was considered. As the pore radius was changed, the relative error associated with it was kept constant.

The signal magnitude as a function of pore size is shown in Figure 7D. The signal change reaches a maximum for the 87 nm pore and then decreases for smaller pore sizes. Figure 7E shows that Jeffreys divergence is low for small pore sizes; however, Jeffreys divergence increases as the pore radius increases. Hence, it is demonstrated that the sensitivity can be adjusted by the choice of pore radius.

### The Applied Voltage Can Alter the Signal Magnitude and Sensitivity

The applied voltage can also influence the variance of the output, with higher applied voltages resulting in broader output distributions (Figure 8A). As the applied voltage increases, the influence of the cone angle random errors also increases (Figure 8B,C). As the signal magnitude increases with increasing applied potential (Figure 8D), the increasing variance is mostly counterbalanced, and sensitivity improves (Figure 8E).

**Figure 8:**
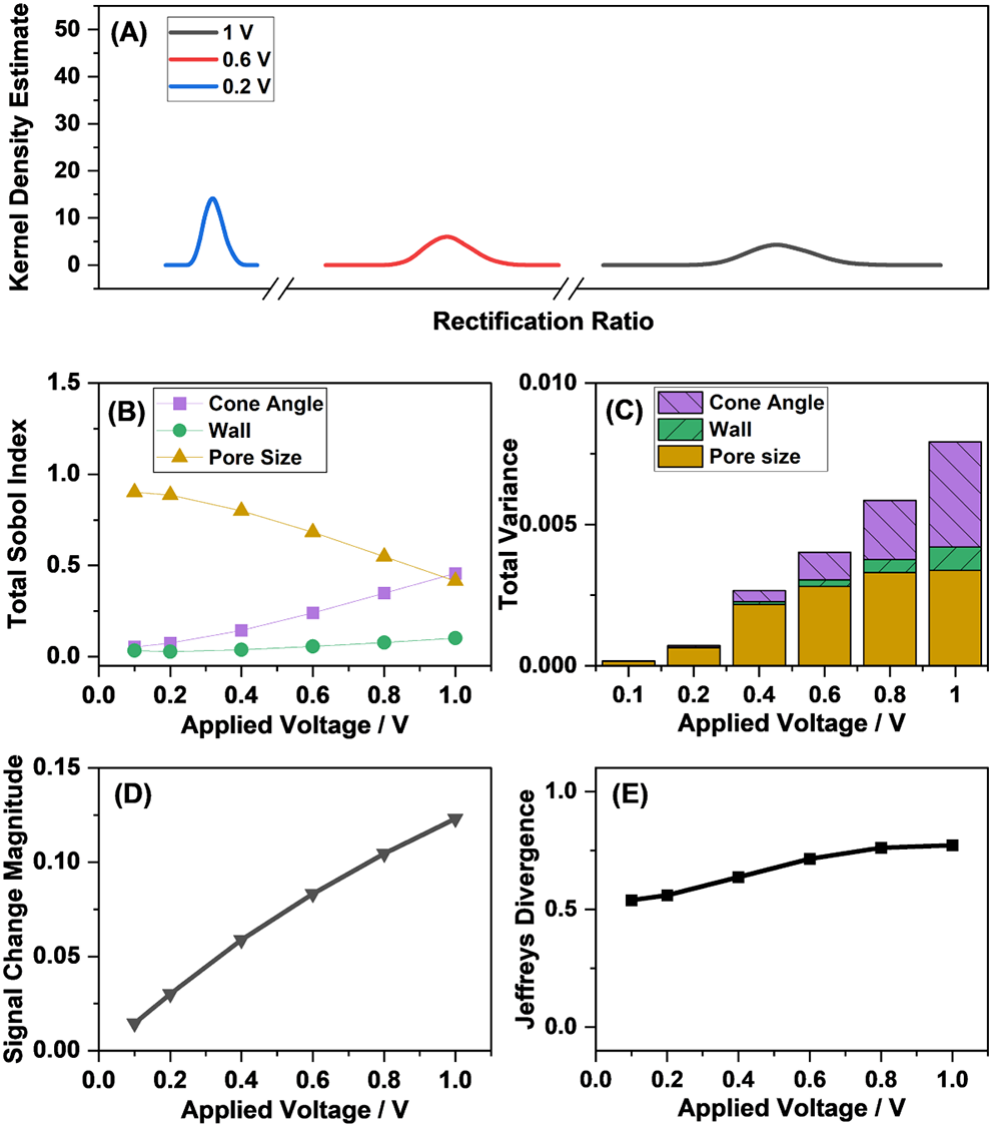
(A) Simulated output distributions of the nanopore in the absence of analyte, (B) Sobol Indices associated with each influential input variable as a function of the applied voltage, (C) total output variance as a function of the applied voltage, (D) simulated signal change magnitude upon the immobilization of 0.01 molecules nm^–2^ analyte, and (E) Jeffreys divergence as a function of the applied voltage. A nanopore with a pore radius of 87 nm and 1 mM electrolyte concentration, 8.5 degree cone angle, and at a temperature of 25 °C was considered.

### The Temperature Can Alter the Signal Magnitude and Sensitivity

Another input variable that can be experimentally controlled is the operating temperature. As shown in Figure 9A,B, unlike changing the pore radius, electrolyte concentration, and the applied voltage, changing the temperature does not have a large effect on the output variance or on the main contributions to the output variance. However, it does have an effect on the magnitude of the signal change, with higher temperatures decreasing the signal (Figure 9C). Consequently, the sensitivity also decreases as the temperature increases (Figure 9D).

**Figure 9:**
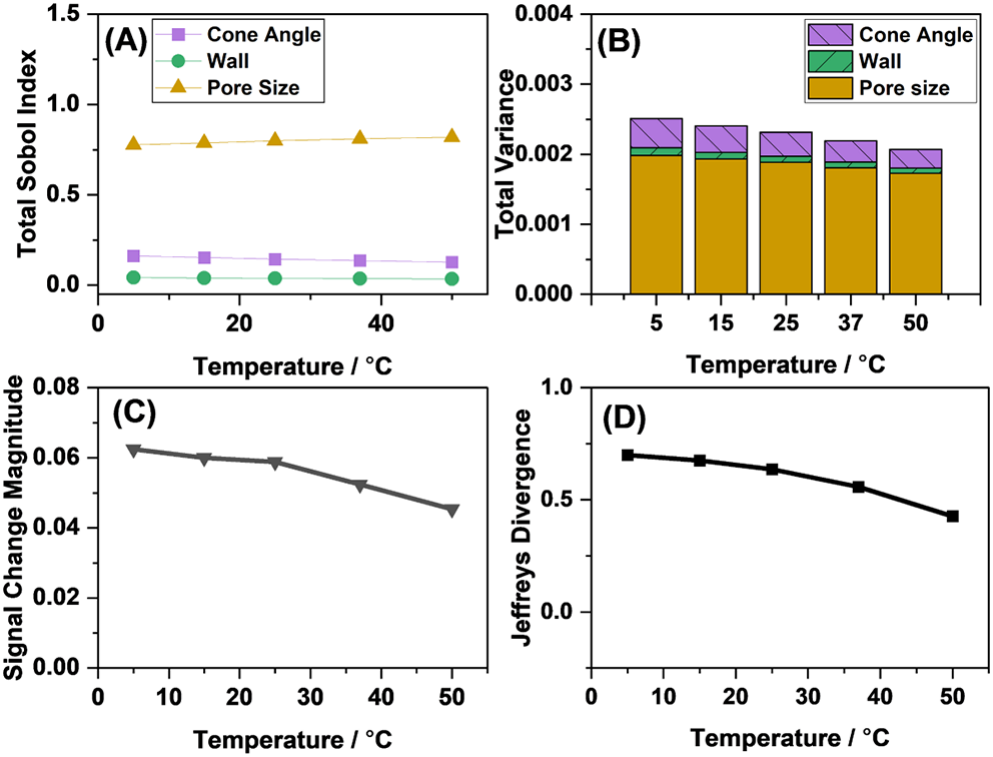
(A) The Sobol Indices associated with each influential input variable as a function of the temperature, (B) total output variance as a function of the temperature, (C) simulated signal change magnitude upon the immobilization of 0.01 molecules nm^–2^ analyte, and (D) Jeffreys divergence as a function of the temperature. A nanopore with 87 nm pore radius and 1 mM electrolyte concentration, 8.5° cone angle, and 0.4 V applied potential was considered.

### Considering Both the Electrolyte Concentration and Pore Size for Sensitivity Optimization

It has been demonstrated that the electrolyte concentration, pore radius, applied voltage, and temperature can all be used to optimize sensitivity by either reducing the influence of random errors on the output variance or increasing the signal magnitude upon analyte immobilization. To identify the highest sensitivity combination of these parameters, all combinations of the four parameters should ideally be investigated, yielding a four-dimensional response surface. However, to mitigate the high computational cost associated with evaluating all permutations of these four parameters, only the pore radius and electrolyte concentration will be considered in the generation of a response surface, as their effects on sensitivity are larger and more nonlinear. In contrast, temperature and voltage influence sensitivity in a consistent direction regardless of pore radius and electrolyte concentration, as shown in Figures S12 and S13. Figure 10 shows the contour plots obtained under the condition where relative errors are kept constant across different pore radii (i.e., errors are considered as a fixed percentage of the pore radius). Essentially, these plots represent the response of the physical phenomena governing transduction only and do not account for the reality that the success of fabricating nanopores with different pore radii varies (resulting in higher or lower relative errors). As such, these plots can serve as a guide for the design parameters of fabrication and provide advice on where experimental efforts in nanopore fabrication should be focused. Figure 10A shows that with the random errors associated with the system, higher sensitivities are expected at larger pore radii and lower supporting electrolyte concentrations. Furthermore, a diagonal trend (indicated by the white dashed line for guidance) can be observed, where the sensitivity maxima for each pore radius shift to higher supporting electrolyte concentrations as the pore radius decreases. This diagonal trend is related to the signal change magnitude, which displays a similar trend (Figure S14); however, the total output variances (Figure S15) are also considered through Jeffreys divergence. Figure 10B shows the Sobol indices as a function of pore radius and electrolyte concentration. These indices can be used to guide experimental efforts, indicating whether resources should be allocated to improving the reproducibility of the cone angle or pore radius during nanopore fabrication.

**Figure 10:**
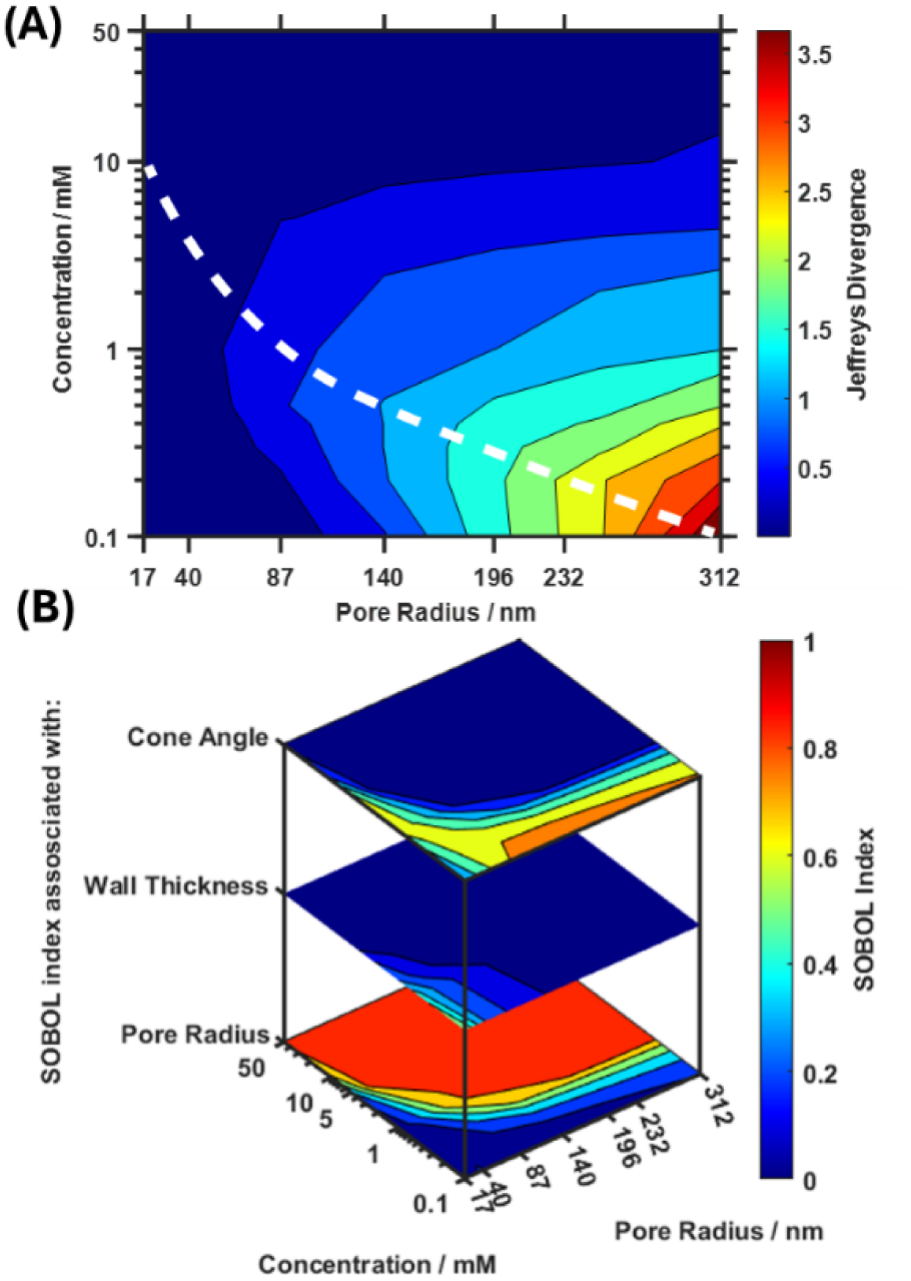
(A) Contour plot of the simulated nanopore sensor sensitivity (Jeffreys divergence) as a function of the supporting electrolyte concentration and pore radius for constant relative errors. The white curve shows the ridge in the contour plot, and this ridge coincides with the ridge of the magnitude of the signal change (Figure S14). (B) Contour plots of the Sobol indices associated with the pore radius, concentration, and wall thickness.

### Real Geometric Errors and Experimental Validation

Once Figure 10 has been utilized to fabricate nanopores of the desired radius, or if existing fabrication methodologies are present, the real errors associated with the fabrication of nanopores with different pore radii can be considered. Figure 11 shows the contour plot obtained when the real geometric errors associated with each pore radius, as determined through STEM analysis (Tables S2), are considered. Here, the same diagonal trend in sensitivity is still observed, with higher sensitivities expected for nanopores with large pore radii at low supporting electrolyte concentrations, as well as the highest sensitivity shifting to higher concentrations as the pore radius is decreased. This trend, which is associated with the behavior of the physical phenomena and the magnitude of the signal change (Figure S14), overlaps with changes to random errors due to the differing quality of fabrication for nanopores with different radii. The sensitivity abruptly changes along the dimension of the pore radius, with higher sensitivities observed for 87 and 232 nm pores due to the higher quality of fabrication for these pore radii in our laboratory, yielding higher reproducibility of the geometric parameters (Figure S16). The 87 nm nanopore has the most reproducible geometric parameters; however, the 232 nm pore has higher overall sensitivity as the signal change magnitude and variance are more optimal. This highlights the importance of simultaneously considering both the physical phenomena that govern the transduction mechanism and the quality of fabrication during the optimization process.

**Figure 11:**
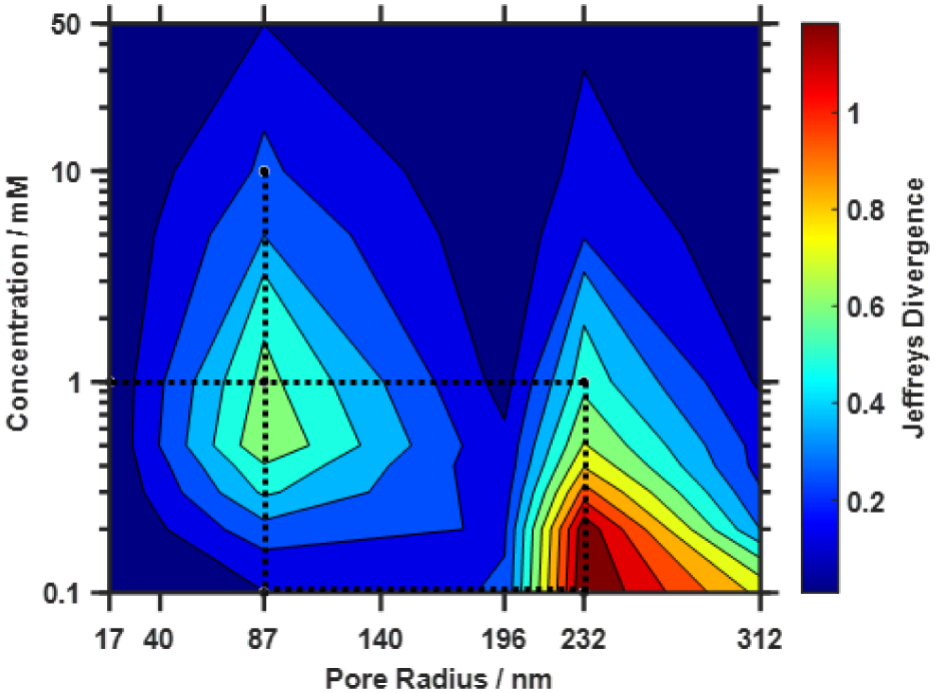
Contour plots of the simulated nanopore sensor sensitivity (Jeffreys divergence) as a function of the supporting electrolyte concentration and pore radius using the real random errors obtained from the fabrication of each pore radius. The highlighted points connected with dashed lines indicate the combinations of concentration and pore radius that were experimentally validated. Sample experimental and simulated current–voltage curves are provided in Figures S7 and S8.

Our model’s ability to predict sensitivity was experimentally validated both as a function of concentration and pore radius. The experimental output distributions of a population of nanopores (at least 60 unique nanopores) in the presence and absence of aminosilane analyte at different pore radii and electrolyte concentrations were collected. These measurements yielded distributions of outputs whose behavior could be compared to those predicted by our model. Figure 12A shows changes to the total output variance as the supporting electrolyte is changed, while Figure 12B shows changes to the total output variance as the pore radius is changed. The total output variance was experimentally observed to decrease as the supporting electrolyte concentration and pore radius were increased. This is in agreement with the simulation results. Although there is qualitative agreement, the magnitude of the predicted variance is always lower for the simulated distributions (note the different axes in Figure 12). This is as expected since in the model, only three parameters are considered to contribute to the variance. The expected rectification ratios are also in very close agreement with those obtained experimentally (Figure S17).

**Figure 12:**
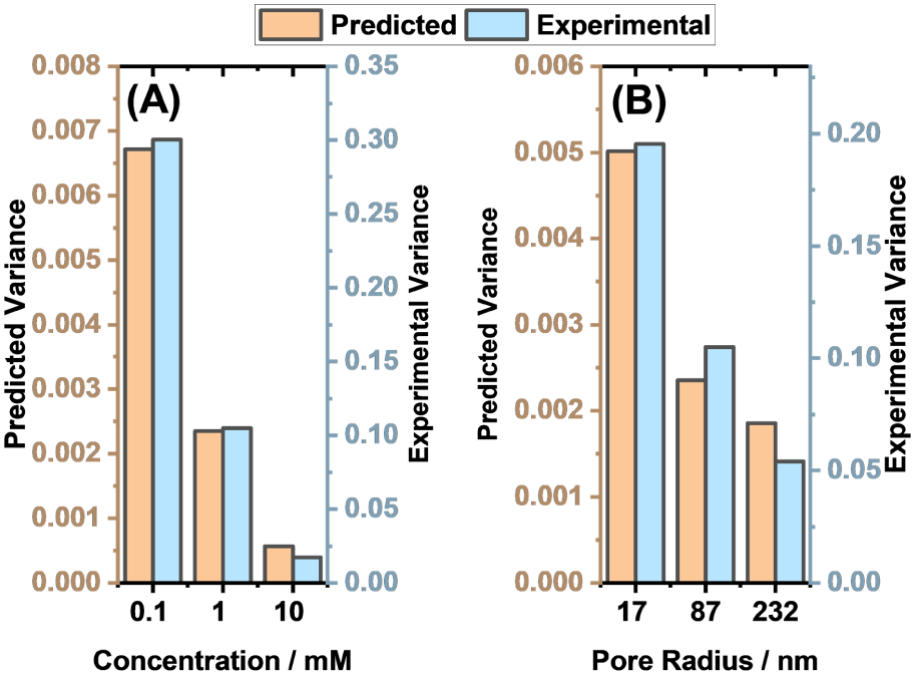
Bar chart comparing experimental and simulated variances of the 87 nm nanopore in the absence of an analyte as the electrolyte concentration is increased and (B) comparing the experimental and simulated variances as the pore radius is increased. As these plots compare to experimental results, the real geometric errors associated with each fabrication are considered. The output distributions are given in Figure S18.

Figure 13 shows the experimentally obtained Jeffreys divergence values for different pore radii and supporting electrolyte concentrations. There is good qualitative agreement with the simulations, with high sensitivities observed for the 232 nm pore at 0.1 mM concentration and for the 87 nm pore at 1 mM concentration, while very low sensitivity is observed for the 17 nm pore at 1 mM concentration. Again, although the qualitative trend is in good agreement, the magnitudes of Jeffreys divergence are different. This is expected, as the experimentally chosen concentration of analyte has no direct linkage to the magnitude of the analyte surface density considered in the model.

**Figure 13:**
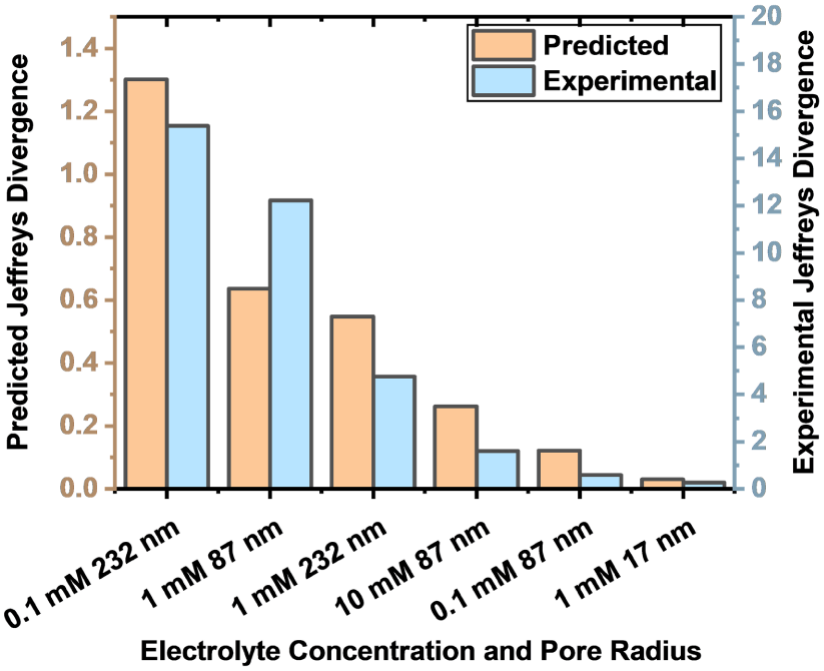
Bar chart comparison of the experimental and simulated Jeffreys divergences for different electrolyte concentrations and pore radii. As these plots compare to experimental results, the real geometric errors associated with each fabrication are considered. The output distributions are given in Figure S18. Data points correspond to the points highlighted and connected in Figure 11. Figure S19 also shows a comparison of the experimental and predicted sensitivities as a function of the applied voltage for the 87 nm pore at a 1 mM electrolyte concentration.

### How the Model Can Be Used to Influence Nanopore Design

Having verified the effectiveness of our model in predicting sensitivity, we now discuss how the results obtained from the model can be applied to provide guidelines on where efforts to improve the reproducibility of nanopore fabrication should be focused. As Sobol indices describe which parameters contribute most to the output variance, improving the reproducibility of parameters with high Sobol indices is the most worthwhile. Furthermore, it is useful to discuss how an improvement in the reproducibility of given parameters will alter the sensitivity map shown in Figure 10.

In general, the influence of cone angle random errors on the variance of the output is greater at lower electrolyte concentrations and pore radii, while the influence of pore radius random errors is greater at higher electrolyte concentrations and pore radii (Figure 10B). As a result, if the nanopore sensor is required to operate at lower electrolyte concentrations and pore radii, it will be most beneficial to focus experimental efforts on improving the reproducibility of the cone angle, whereas a nanopore operated at high electrolyte concentrations and pore radii will benefit most from improved pore radius reproducibility. However, utilizing extremely low pore radii and concentrations, or extremely high pore radii and concentrations, is detrimental as the signal change magnitude is lost. This is indicated by the curvature highlighted in Figure 10. This curvature arises due to the interplay between the signal change magnitude (Figure S14) and the output variances contributed by the pore radius, cone angle, and wall thickness (Figure 10B). As the magnitude of the signal change is related to the physical phenomena governing signal transduction, it is unaffected by changes to the reproducibility of the pore radius or the cone angle. However, such improvements do affect the total contributed variance. The curvature of the ridge in Figure 10A will shift to lower concentrations and pore radii if the reproducibility of the cone angle is improved, and it will shift to higher concentrations and pore radii if the reproducibility of the pore radius is improved. This is useful to understand when designing a sensor that may be constrained to certain pore radii or electrolyte concentrations.

We will propose a series of experimental scenarios that reflect work with ion-current-rectifying nanopores, suggest optimal pore geometries and experimental conditions, and draw more specific conclusions on which aspect of fabrication is most worthwhile to improve. For all scenarios, increasing the applied voltage and decreasing the temperature will be advantageous (Figures S12 and S13). Considerations of the pore radius and electrolyte concentration are more nuanced. Figure S20 is a marked-up version of Figure 10A providing the reader with a visual highlight of each scenario’s recommended pore radius and electrolyte concentration.

### Scenario 1: Nanopipette Sensors with Large Pore Radii for the Analysis of Large Analytes Such as Nanoparticles and Proteins

With a limitation on using larger pore radii, the electrolyte concentration should be decreased as much as possible to achieve optimum sensitivity. The significance of random errors in the pore radius increases with larger pore radii; however, the significance of random errors in the cone angle increases with the use of low electrolyte concentrations. As such, both the cone angle and the pore radius are influential contributors to the variance. The cone angle has a greater contribution; therefore, sensitivity could be most improved if the cone angle errors are addressed by an improved fabrication method (Figure 10B).

### Scenario 2: A Nanopipette Sensor for Use in High Salt Conditions, for Example, in the Analysis of Biological Fluids

With the limitation of using high supporting electrolyte conditions, using small pore radii will be beneficial (Figure S20) mainly because the signal change magnitude at high concentrations improves as the pore radius is decreased (Figure S14). Despite the low pore radius, at these high concentrations, the most important source of variance is the pore radius; as such, further improvements in sensitivity could be gained by improving the reproducibility of the pore radius (Figure 10B).

### Scenario 3: Nanopipettes for Localized Measurements, Such as at Surface-Bound Catalytic Sites, and at Individual Pores of Porous Materials or Membranes

In this scenario, the viable nanopore radii are limited to low values to retain spatial resolution. With a limitation on the viable nanopore radii, the concentration should be considered. Intermediate concentrations around 10 mM are expected to show the highest sensitivity (Figure S20). Under these conditions, both pore radii and cone angle random errors contribute to the output variance. As such, if improvement and optimization of the fabrication methodology are viable, either of these factors can be addressed to further improve sensitivity (Figure 10B). It should be noted that in the case of localized measurement, there are additional considerations that may need to be considered such as possible approach distances and the nature of the localized point source of analyte. For a comprehensive understanding of sensitivity in localized measurements, the model should be extended to include these.

### Scenario 4: A Nanopipette Sensor for In Vitro Cell Measurements under Physiological Salt Conditions

For this experimental design, the salt concentration is high, while the nanopore diameter must be low due to the requirement of penetrating the cell membrane. As both the concentrations and the pore radius are limited, improvements in sensitivity need to be approached from a fabrication point of view. The most important source of variance will be the pore radius (Figure 10B); as such, this should be addressed. For our modeled systems, the ideal sensitivity for the small pore radii was around 5–10 mM, which is still below physiological salt conditions. An improvement in the pore radius reproducibility would shift this to higher electrolyte concentrations, closer to those observed at physiological salt concentrations.

### Limitations of the Model

Although good agreement was obtained between simulations and experiments, there are limitations in the model that need to be highlighted. Specifically, there are sources of random errors that are not taken into account, and hence, the simulated output distributions are narrower than those observed in experiments. Sources of variance not considered include the extent of analyte immobilization and variation in the number of available Si–OH surface groups on the quartz surface. The incorporation of these factors would be computationally straightforward; however, obtaining reasonable values for these uncertainties would require extensive characterization of the internal walls of the nanopore. For these nanopore systems, this would be highly challenging, which is why the assumptions used in this work were made. Other limitations include the simplification of some physical phenomena, which could introduce additional complexity. For example, the extent of analyte adsorption is assumed to be the same regardless of the pore size, electrolyte concentration, and temperature considered; however, the equilibrium reactions of surface adsorption are influenced by these parameters. Additionally, the surface charge density is known to vary along the longitudinal direction of the nanopore;^57^ however, this detail is simplified by considering a constant surface charge density. The incorporation of these physical phenomena would make the prediction of sensor sensitivity more accurate, albeit at the cost of higher modeling complexity. Another physical simplification of the model that must be highlighted is the omission of the Navier–Stokes equations due to the high computational cost. The Navier–Stokes equations are used to describe fluid flow and electroosmotic flow, which are known to play an important role in the behavior of nanopores, especially at moderate electrolyte concentrations where both the diffuse layer and the surface charge density are large.^45,64,65^ The inclusion of the Navier–Stokes equations is expected to be especially relevant for identifying the influences on the output variance and sensor sensitivity as a function of variables that have a strong coupling with fluid flow. An example would be temperature, which alters the viscosity of the solution and hence could result in larger and more complex influences than reported herein. Additionally, the fluctuation of current (mid-measurement noise) is not considered, as it is more relevant to time-dependent measurements, such as particle translocation. The rectification ratio, however, is a steady-state metric; as such, instrument noise does not have a significant impact on the extracted average current. In addition to the simplification of physical phenomena, computational simplifications also need to be highlighted. The surface charge densities are calculated in a separate model (they are decoupled); hence, the distributions of the pore radii, cone angle, and wall thickness are not propagated to a distribution in the surface charge density. Although the full coupling of the input parameter distributions and corresponding surface charge densities can be implemented, the computational cost of each evaluation would increase significantly. The inclusion of this coupling would lead to simulated outputs with broader distributions, which would be closer in magnitude to those obtained experimentally. Regardless of the limitations discussed here, we emphasize that in its current form, the model remains in good agreement with the experimental data.

## Conclusion

To conclude, finite element simulation is used to demonstrate how the sensitivity of nanopore sensors can be optimized by adjusting the geometric and operating conditions, namely the pore radius, electrolyte concentration, applied voltage, and temperature. The influence of these parameters on the magnitude of the signal change and the output variance (arising from the propagation of random errors) was investigated. As the pore radius and electrolyte concentration are decreased, the influence of random errors arising from the cone angle increases, and the output becomes broader. The signal magnitude reaches a maximum at intermediate pore radii and electrolyte concentrations. The sensitivity of the sensor was found to be greater at larger pore radii operated at low electrolyte concentrations; however, particularly good fabrication methodologies can result in high sensitivities under other conditions as well. Good experimental agreement validates the simulations. The sensitivity optimization presented herein is, in principle, viable for any nanoscale sensor whose transduction mechanism operates at a scale that can be modeled by continuum physics. The FEA-based optimization presented here is expected to greatly assist the research and development of nanoscale devices by minimizing the need for tedious experimental optimization.

## Supplementary Material



## Notes

Description of the finite element model, derivation of surface charge equations, sample surface charge densities, description of temperature-dependent parameters, sample STEM images, Morris plots under different conditions, effects of temperature and voltage at additional conditions, effects of the cone angle, sample experimental current–voltage curves, experimental distributions, rectification ratio values, fabrication errors, and multidimensional plots of the variance and signal change magnitude (PDF)
