# Supplementary material for: Understanding Sensitivity in Nanoscale Sensing Devices
Source: ACS Meas Sci Au. 2025 Apr 15;5(3):353–66. doi: 10.1021/acsmeasuresciau.5c00023 (PMC12183584; doi:10.1021/acsmeasuresciau.5c00023)
Supplement: Supplementary file 1 [file tg5c00023_si_001.pdf]

Understanding Sensitivity in Nanoscale Sensing Devices

Dominik Duleba*, Adria Martínez-Aviñó, Andriy Revenko, Robert P. Johnson*

School of Chemistry, University College Dublin, Belfield, Dublin 4, Ireland

*dominik.duleba@ucdconnect.ie, robert.johnson@ucd.ie

Table of Contents

COMSOL Model 2

Surface Charge Density Expressions 3

Sample of Surface Charge Densities for the 87 nm Pore as a Function of Concentration 6

Considering the Temperature Dependent Parameters 7

STEM Images 10

Sample Experimental Current-Voltage Curves 12

Simulated Current-Voltage Curves 13

Morris Plots at Different Pore Radii and Concentrations 14

The Effect of the Cone Angle 15

The Effect of Temperature at Different Pore Radii and Electrolyte Concentrations 17

The Effect of Voltage at Different Pore Radii and Electrolyte Concentrations 18

Signal Change Magnitude Surface 19

Total Variance and Contributions 20

Total Percentage Errors for Visualization of Fabrication Quality 21

Agreement of Experiments with the RR Value 22

Experimental Distributions of the Rectification Ratio 23

Experimental Jeffreys Divergence as a Function of the Applied Voltage 24

Recommended Operating Conditions for Different Scenarios 25

References 26

COMSOL Model

The model's geometry consists of a 10 µm long conical pipette and a 2 µm wide bulk solution represented in a 2D-axisymmetric geometry. Both the pipette length and the bulk solution size have been verified to be large enough for the solutions not to be affected.[1]

The model is meshed using a very fine mesh near the nanopore tip and near charged surfaces, while larger elements are utilized in the bulk solution. (Figure S1) Boundary layer type elements are used on the charged surfaces, while elsewhere triangular elements are used. Mesh refinement studies show that further reducing the element sizes does not change the solution. The integrated ion fluxes are consistent across different boundaries indicating flux continuity. The final mesh used contains around 180000 elements and the model solves with around 1800000 (plus 110000 internal degrees of freedom).

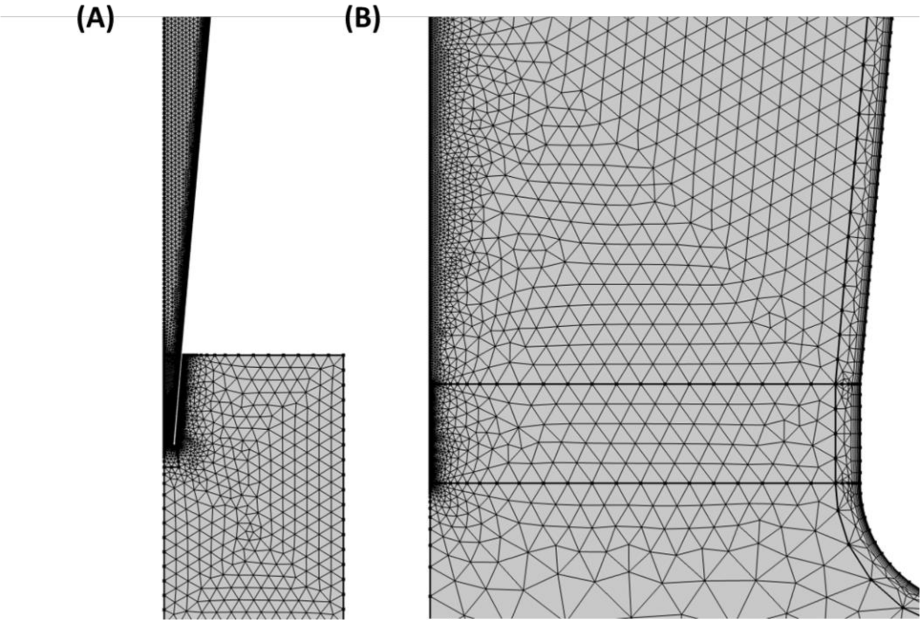

Figure S1 (A) Image of the meshing throughout the nanopore, and (B) a zoomed in image of the meshing the nanopore tip.

Figure S2 shows the boundary conditions used for the model. A surface charge density boundary conditions is applied to the quartz surface the nanopipette. The magnitude of the surface charge density is decreased using a step function near the concentration boundaries. This aids model convergence and flux continuity. In the exterior bulk solution, only one boundary has the concentration/applied potential boundary applied (with the other two boundaries having a no-flux and zero charge boundary condition). This is done so that the

boundary integrated for the ionic flux is not bordering a surface charge density boundary, which could lead to incorrect integrated ion fluxes. The no-flux boundaries are sufficiently far from the nanopore mouth and does not affect ion transport.

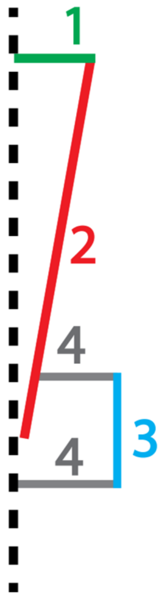

Boundary no.	Boundary Description	Nernst-Planck eq.	Poisson eq.
1	Internal bulk solution	Constant Concentration $[K^+]$ and $[Cl^-]$	Constant Potential (applied) $V = volt$
2	Pipette walls	No Flux $\boldsymbol{n} \cdot \nabla \boldsymbol{J}_i = 0$	Surface Charge $\boldsymbol{n} \cdot \nabla \Phi = -\frac{\sigma}{e}$
3	External bulk solution	Constant Concentration $[K^+]$ and $[Cl^-]$	Constant Potential (ground) $V = 0$
4	Bulk solution walls	No Flux $\boldsymbol{n} \cdot \nabla \boldsymbol{J}_i = 0$	Zero Charge $\boldsymbol{n} \cdot \boldsymbol{D} = 0$

Figure S2 shows the boundary conditions applied to different parts of the nanopore geometry.

The model incorporates the Poisson equations and the Nernst-Planck equations twice. One pair of the Poisson-Nernst-Planck equations solves for the positive applied potential, while the other pair solves for the negative applied potential. This is a necessity as the rectification ratio must be evaluated in a single model evaluation, so that the uncertainty quantification node can then solve for a different sample of the input parameter distributions.

Surface Charge Density Expressions

An accurate description of the surface charge density and its change upon analyte immobilization is crucial to predict the sensor sensitivity at any given set of operating conditions with good validity. The surface charge density of the surface is described based on the density of relevant surface groups and their equilibrium constants.

Taking the relevant equilibrium reactions with equilibrium constant K_1, K_2 and K_3 respectively:

$$SiOH + H_2O \leftrightarrow SiO^- + H_3O^+$$

$$SiOH_2^+ + H_2O \leftrightarrow SiOH + H_3O^+$$

$$NH_3^+ + H_2O \leftrightarrow NH_2 + H_3O^+$$

The surface charge of the surface is determined by the number of SiO^-, $SiOH_2^+$, $NH_3^+$and present. For the surface charges contributed by the quartz silanol groups:

$$\sigma = -e(\Gamma_{SiO^-} - \Gamma_{SiOH_2^+}) \qquad \text{Eq. S1}$$

where Γ_j denotes the surface density of species j in molecules/m^2.

Expressions of surface density can be obtained by rearranging the equilibrium constant equations, where the activities of water are taken as unity, arriving at:

$$\Gamma_{SiO^-} = K_1 \frac{\Gamma_{SiOH}}{[H_3O^+]} \qquad \text{Eq. S2}$$

$$\Gamma_{SiOH_2^+} = \frac{\Gamma_{SiOH}[H_3O^+]}{K_2} \qquad \text{Eq. S3}$$

where $[H_3O^+]$ denotes the molar proton concentration in the element adjacent to the charged surface.

Substituting Eq. S2 and S3 into Eq. S1:

$$\sigma_{silanol} = -e\left(K_1 \frac{\Gamma_{SiOH}}{[H_3O^+]} - \frac{\Gamma_{SiOH}[H_3O^+]}{K_2}\right) \qquad \text{Eq. S4}$$

Now to express Γ_{SiOH} in terms of the known variable $[H_3O^+]$ and constants, we recognize that Γ_{SiOH} is related to the total surface density of surface silanol groups:

$$\Gamma_{SiOH} = \Gamma_{tot,silanol} - \Gamma_{SiO^-} - \Gamma_{SiOH_2^+} \qquad \text{Eq. S5}$$

Substituting in Eq 2 and 3:

$$\Gamma_{SiOH} = \Gamma_{tot,silanol} - K_1 \frac{\Gamma_{SiOH}}{[H_3O^+]} - \frac{\Gamma_{SiOH}[H_3O^+]}{K_2} \text{ Eq. S6}$$

Factorizing and rearranging for Γ_{SiOH}:

$$\Gamma_{SiOH} = \frac{\Gamma_{tot,silanol}}{1 + \frac{K_1}{[H_3O^+]} + \frac{[H_3O^+]}{K_2}} \qquad \text{Eq. S6}$$

Finally, substituting Eq. S6 into the surface charge expression (Eq. S4) gives the final form of the equation describing the surface charge density arising from the protonation/deprotonation of silanol groups. The surface charge density is a function of constants and of the local proton concentration which is a variable in the finite element analysis.

$$\sigma = -e\Gamma_{tot,silane}\left(\frac{\frac{K_1}{[H_3O^+]}-\frac{[H_3O^+]}{K_2}}{1+\frac{K_1}{[H_3O^+]}+\frac{[H_3O^+]}{K_2}}\right) \qquad \text{Eq. S7}$$

Next, the surface charge contribution of the amine group can be considered similarly, and added to Eq. S7 with a consideration that the amine groups are replacing some of the quartz silanol groups:

$$\sigma = -e\left(\Gamma_{tot,silanol} - \Gamma_{tot,amine}\right)\left(\frac{\frac{K_1}{[H_3O^+]}-\frac{[H_3O^+]}{K_2}}{1+\frac{K_1}{[H_3O^+]}+\frac{[H_3O^+]}{K_2}}\right) - e\Gamma_{tot,amine}\left(\frac{[H_3O^+]}{K_3}\right) \quad \text{Eq. S8}$$

Sample of Surface Charge Densities for the 87 nm Pore as a Function of Concentration

The expression of surface charge density used herein is important as both the surface charge density and the change in the surface charge density upon analyte immobilization are strongly dependent on the sensor's operating conditions. To illustrate this, Table S1 provides the surface charge densities and the percentage change upon analyte immobilization as a function of the electrolyte concentration.

Table S1 Surface Charge densities of the 87 nm pore at 0.4 V applied voltage magnitude and room temperature, as a function of the electrolyte concentration.

Electrolyte Concentration / mM	Unmodified Surface Charge Density / mC m^{-2}	Modified Surface Charge Density / mC m^{-2}	Percentage Change in Surface Charge Density
50	-17.299	-16.605	-4
10	-10.694	-10.108	-5
5	-8.4226	-7.8794	-6
1	-4.6401	-4.1521	-11
0.5	-3.6018	-3.1234	-13
0.1	-2.0687	-1.6137	-22

Considering the Temperature Dependent Parameters

To consider the temperature dependence of the sensitivity, as well as to correctly calculate the surface charge densities of the modified and unmodified nanopores, the temperature dependence of parameters must be included in the simulations. The relevant temperature dependent parameters are the diffusion coefficients, the dielectric constant, and the acidity constant values.

The values of the diffusion coefficients at different temperatures are estimated using the limiting ionic conductance of the ion:[2]

$$D_j = \frac{RT\,\Lambda_j^0}{|z_j|F^2}$$

where D_j is the diffusion coefficient at the given temperature, Λ_j^0 is the limiting ionic conductance at the given temperature, and z_j is the charge number of the ion. The limiting ionic conductance data as a function of temperature was obtained from Robinson and Stokes.[3] Figure S3 shows the diffusion coefficients of K^+, Cl^-, H^+ and OH^- as a function of temperature.

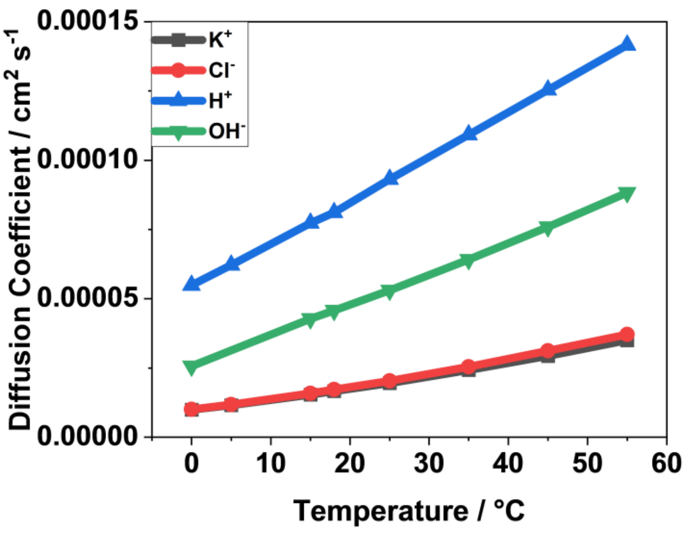

Figure S3 The diffusion coefficients as a function of temperature as estimated from each ion's limiting ionic conductance.

The values of the dielectric constant at different temperatures are from Malmberg and Maryott (Figure S4).[4]

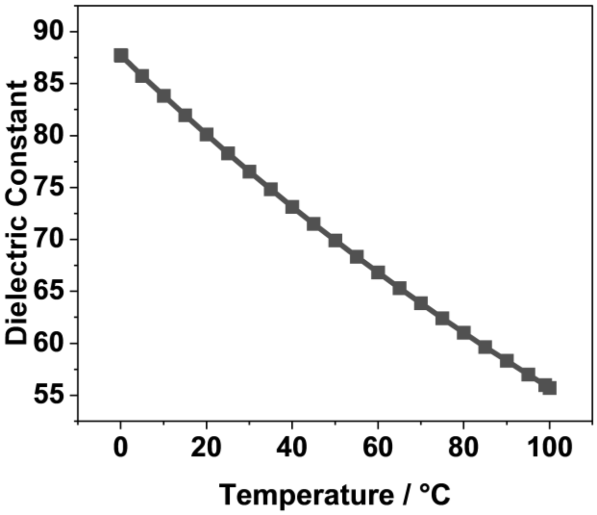

Figure S4 The dielectric constant as a function of temperature

The values of the acidity constant at different temperatures are estimated using the Van ’t Hoff equation:

$$\ln\frac{K_{T_x}}{K_{RT}} = \frac{\Delta_r H^0}{R}\left(\frac{1}{T_{RT}} - \frac{1}{T_x}\right)$$

where K_x is the equilibrium constant at temperature T_x, K_{RT} is the equilibrium constant at room temperature, T_{RT} is room temperature, and $\Delta_r H^0$ is the enthalpy change of protonation. The enthalpy change is assumed constant between temperatures. The enthalpy change of protonation is taken as 2 kJ/mol and 21 kJ/mol for the quartz silanol protonation reactions K_1 and K_2.[5] For the surface-bound amine group, the enthalpy change n-butylamine is used, with a value of 58 kJ/mol.[6] For the acidity constant values of the quartz silanol groups at room temperature, pK_1 is given the value of 8 while pK_2 is given the value of 0.5, both of which are consistent with literature values used elsewhere.[7–13] For the immobilized amine, the value of 7.4, which was obtained from the force titration an aminosilane, is used.[14] The acidity constant for each protonation reaction as a function of temperature are shown in Figure S5.

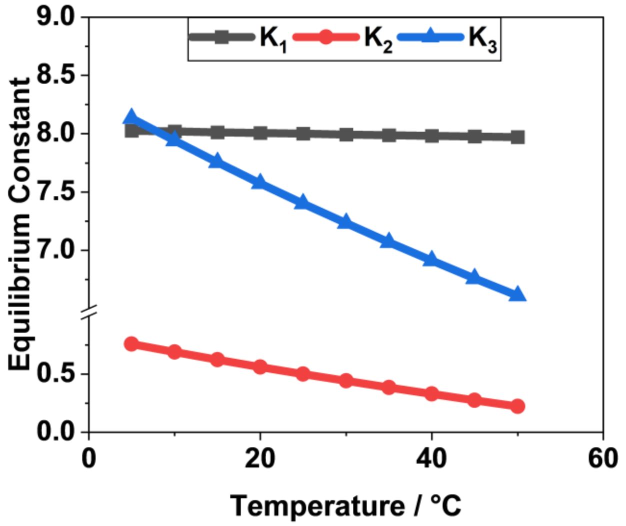

Figure S5 The equilibrium constants as a function of temperature.

STEM Images

To obtain valid distributions of the sensor output, the distributions of the input variables must be as representative as possible. As the most influential input variables are geometric, scanning transmission electron microscopy (STEM) imaging was used to obtain the average and standard deviation of pore radius, cone angle and wall thickness. At least 6 individual pipettes from each fabrication program were imaged to obtain the average and standard deviation values. Figure S6 provides representative images, while Table S2 provides the averages and standard deviations of the geometric parameters.

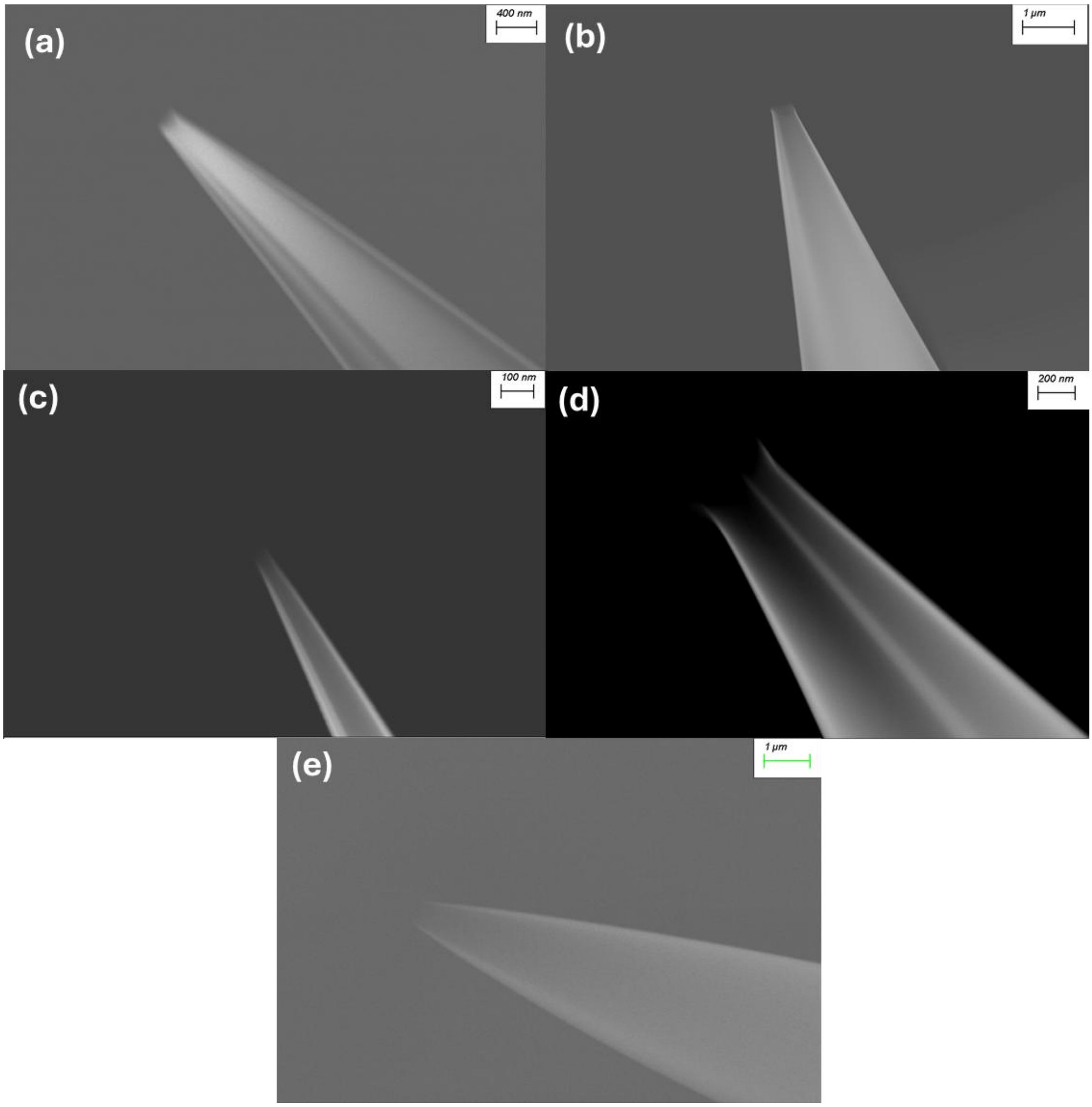

Figure S6 shows representative STEM images of the pores with average radii of (a) 87 nm, (b) 196 nm, (c) 17 nm, (d) 312 nm, and (e) 232 nm.

Table S2 The mean and standard deviations ($\bar{X} \pm S$) of the geometric parameters extracted from STEM analysis.

Program	Pore Radius / nm	Cone angle / degree	Wall Thickness / nm
H750 F4 V40 D135 P180	17 ± 3	4.1 ± 0.4	8 ± 2
Line 1: H700 F4 V20 D170 P0,<br>Line 2: H680 F4 V50 D170 P200	87 ± 7	8.5 ± 0.8	27 ± 7
H580 F3 V55 D128 P110	196 ± 52	10 ± 2	30 ± 20
Line 1: H610 F4 V55 D150 P80,<br>Line 2: H560 F3 V30 D135 P103	232 ± 27	11 ± 1	56 ± 8
H575 F3 V60 D128 P100	312 ± 77	9.6 ± 0.8	50 ± 30

Sample Experimental Current-Voltage Curves

For the experimental validation of the model presented herein, current-voltage curves in the presence and absence of an aminosilane analyte were recorded. The immobilization of the aminosilane contributes a positive surface charge density to the surface, as such the rectification ratio decreases. A representative pair of current-voltage curves in the presence and absence of analyte for different pore radii and electrolyte concentrations are provided in **Error! Reference source not found.**.

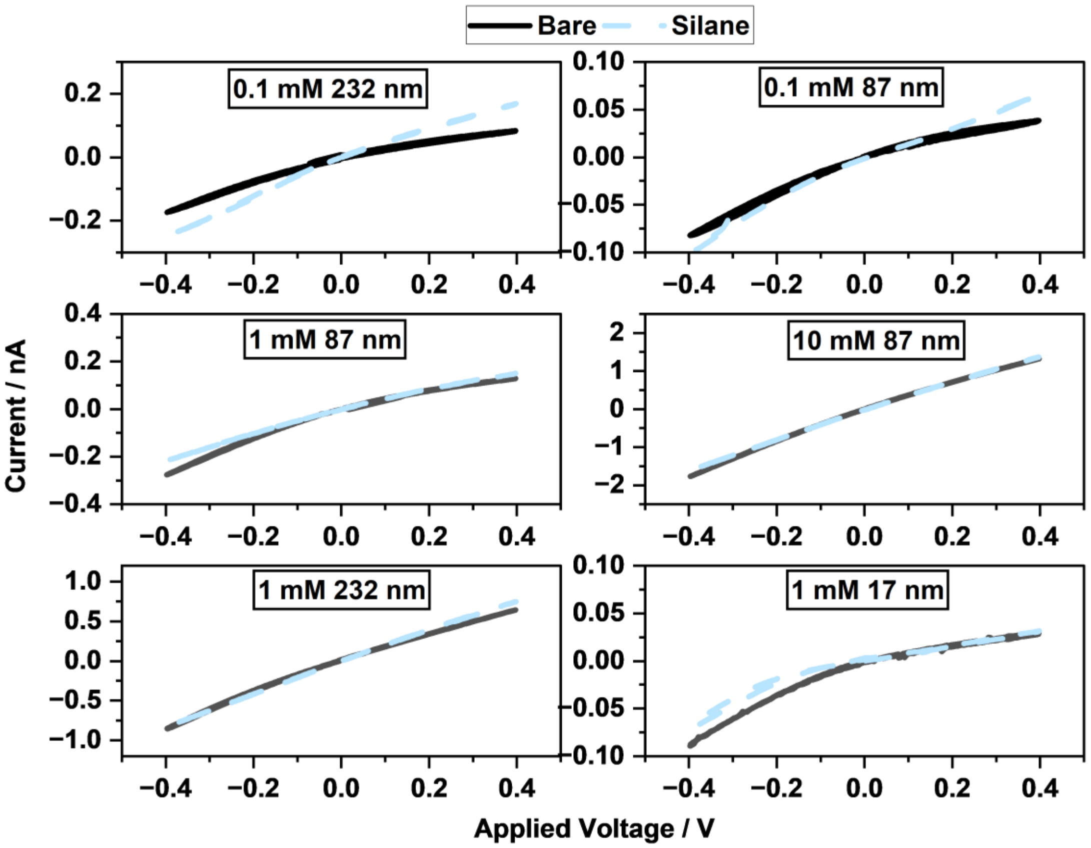

Figure S7 Sample experimental current-voltage curves for all combinations of pore radii and concentrations that were used for experimental validation.

Simulated Current-Voltage Curves

The simulated current-voltage curves provided in Figure S8 show good agreement with the experimental current-voltage curves presented in Figure S7. The shapes and current magnitudes of the unmodified current-voltage curves, as well as the qualitative direction of change and shape of current-voltage curves upon silane modification are in agreement. The quantitative magnitude of change is greater in experiment, as the model made no attempts at quantifying the magnitude of change, rather the aim was to qualitatively compare the change at different conditions (as also explained in the main paper).

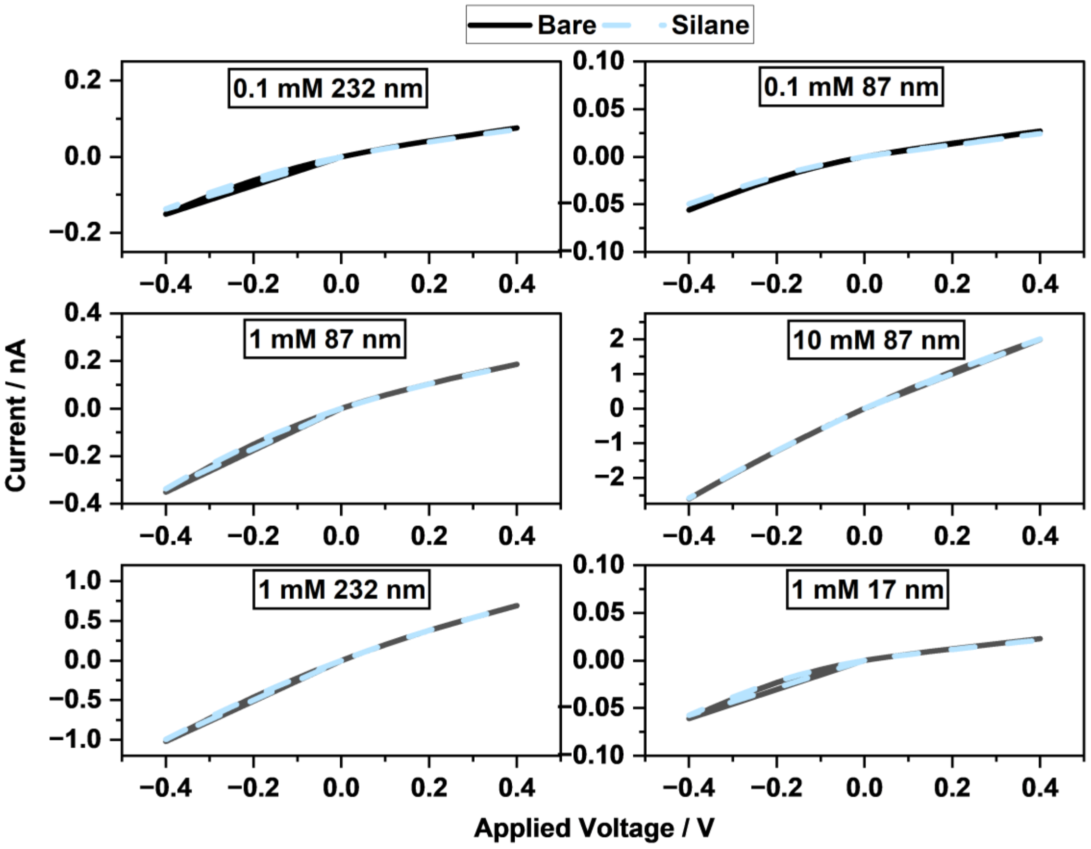

Figure S8 Simulated current-voltage curves for all combinations of pore radii and concentrations that were used for experimental validation. The simulated current-voltage curves can be compared to the experimental current-voltage curves in Figure S7.

Morris Plots at Different Pore Radii and Concentrations

As the influence of the input variable random errors on the output distribution depends on the operating conditions, in addition to the Morris plot provided in the main paper, Figure S9 provides the Morris plots for a nanopore with a larger pore radius and for a nanopore with a lower electrolyte concentration.

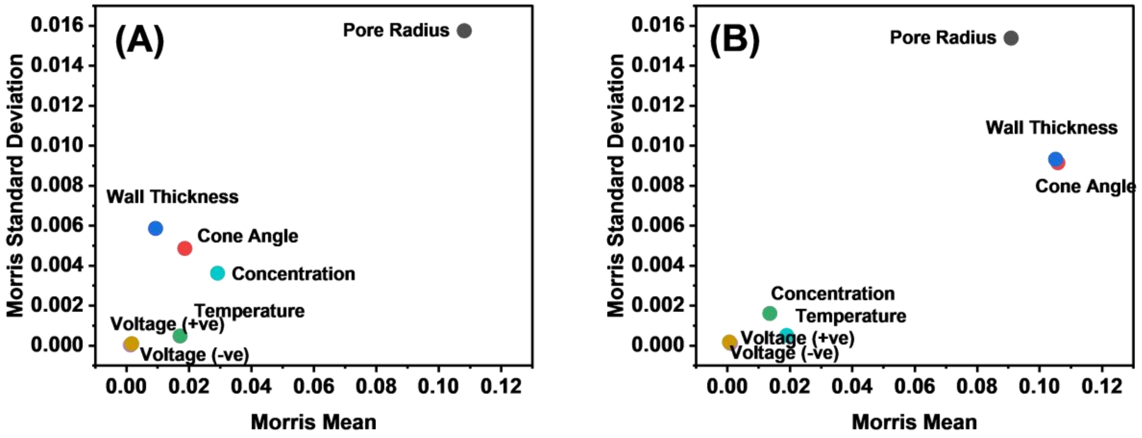

Figure S9 Morris plot for an (A) for a 312 nm nanopipette at 1 mM electrolyte concentration and (B) 87 nm nanopipette at 0.1 mM electrolyte concentration.

The Effect of the Cone Angle

Although for our nanopore systems, control of the cone angle is not feasible, for different nanopore fabrication procedures cone angle control may be feasible. As such, the effect of the cone angle on the Sobol indices, the signal change, and the Jeffreys Divergence are provided in Figure S10. The effect of the cone angle is also provided for a nanopore with a larger pore radius and for a nanopore with a lower electrolyte concentration in Figure S11

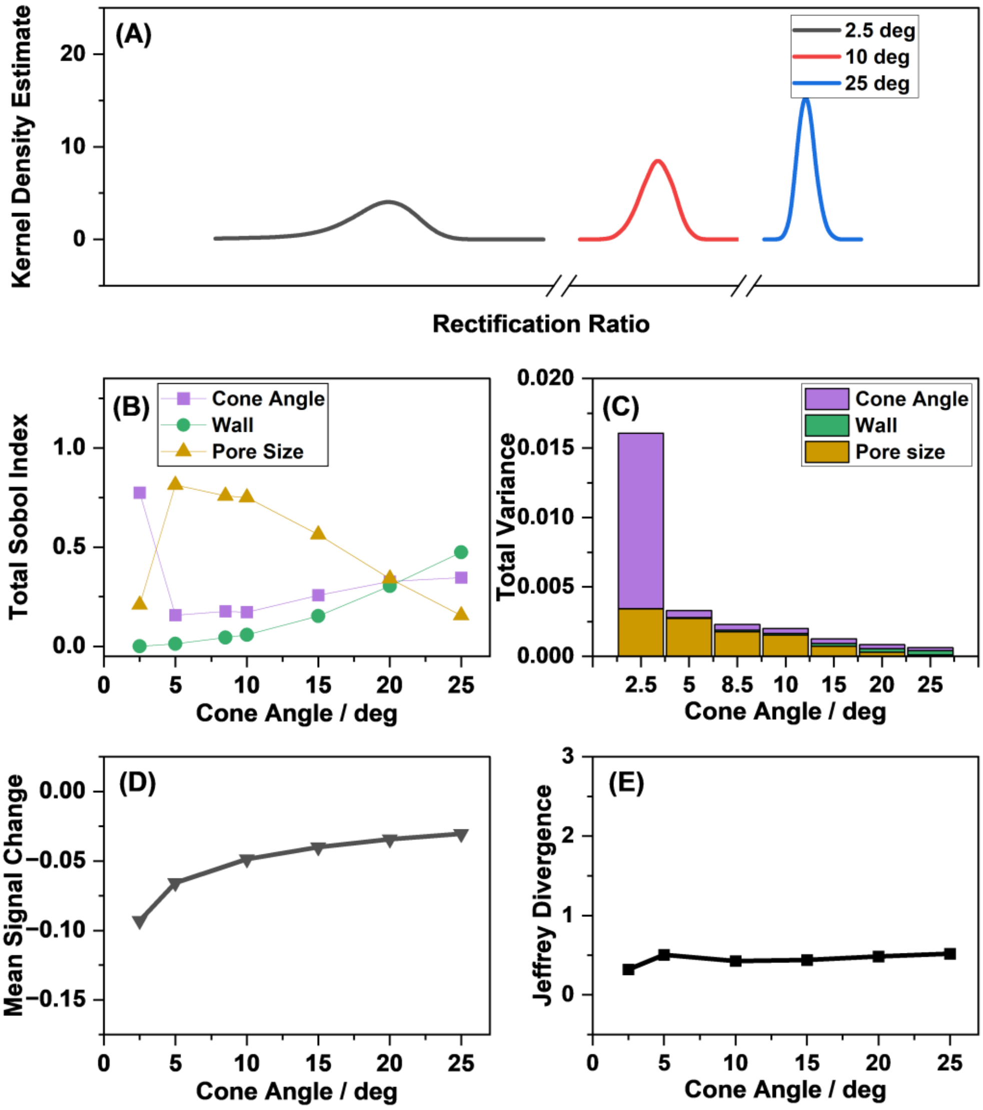

Figure S10 (A) Simulated output distributions of the nanopore in the absence of analyte, (B) the Sobol Indices associated with each influential input variable as a function of the internal cone angle, (C) simulated total output variance as a function of the internal cone angle, (D) simulated signal change magnitude upon the immobilization of 0.01 molecules nm^{-2} analyte, and (E) Jeffreys Divergence as a function of the internal cone angle. A nanopore with at 1 mM electrolyte concentration, 87 nm pore radius, at 25 °C and 0.4 V applied potential was considered.

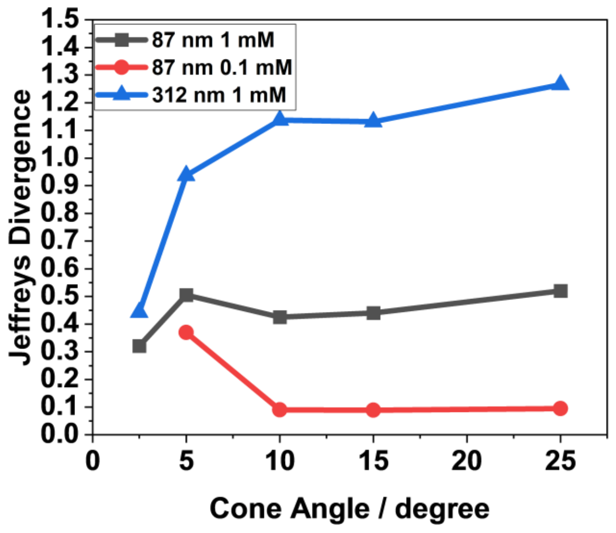

Figure S11 The simulated Jeffreys Divergence as a function of the internal cone angle for a different pore radius and electrolyte concentration.

The Effect of Temperature at Different Pore Radii and Electrolyte Concentrations

The main paper presents the effect of temperature on the sensitivity for a nanopore with an 87 nm pore radius at 1 mM electrolyte concentration. Here, the effect of temperature is also provided for a nanopore with a larger pore radius and for a nanopore with a lower electrolyte concentration.

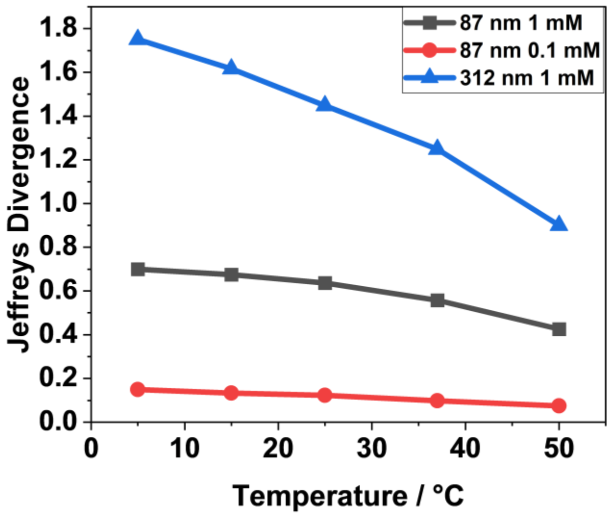

Figure S12 Simulate Jeffreys Divergence as a function of the temperature for a different pore radius and electrolyte concentration.

The Effect of Voltage at Different Pore Radii and Electrolyte Concentrations

The main paper presents the effect of the applied voltage on the sensitivity for a nanopore with an 87 nm pore radius at 1 mM electrolyte concentration. Here, the effect of the applied voltage is also provided for a nanopore with a larger pore radius and for a nanopore with a lower electrolyte concentration.

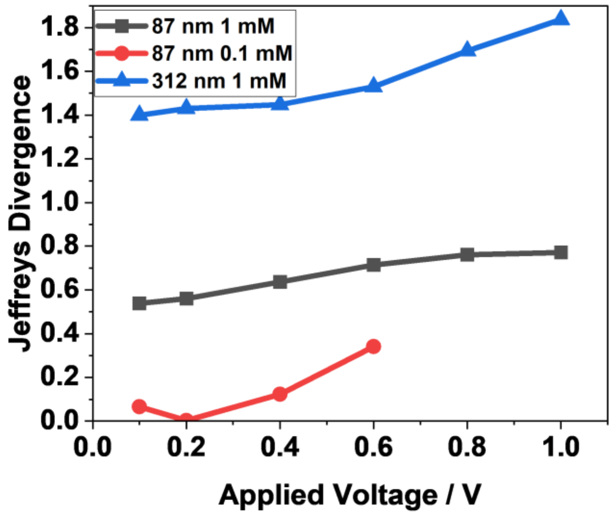

Figure S13 Simulated Jeffreys Divergence as a function of the applied voltage for a different pore radius and electrolyte concentration.

Signal Change Magnitude Surface

In addition to the signal change magnitude for the 87 nm pore radius as a function of electrolyte concentration (as presented in Figure 4), and for 1 mM electrolyte concentration as a function of the pore radius (as presented in Figure 6), **Error! Reference source not found.** the signal change magnitude at each studied pore radius and electrolyte concentration.

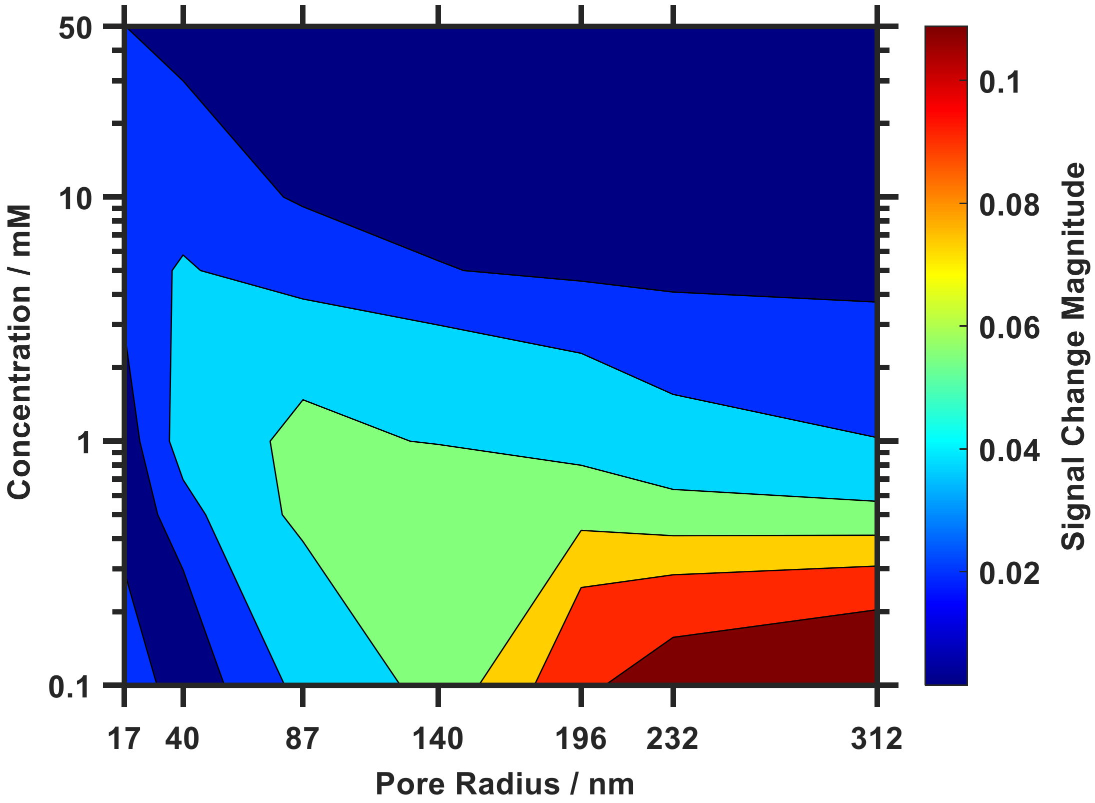

Figure S14 shows the simulated signal change magnitude as a function of both the electrolyte concentration and pore radius.

Total Variance and Contributions

In addition to the total output variance for the 87 nm pore radius as a function of electrolyte concentration (as presented in Figure 4), and for 1 mM electrolyte concentration as a function of the pore radius (as presented in Figure 6), Figure S15 presents the total variance and contributions at all studied combinations of pore radius and electrolyte concentration.

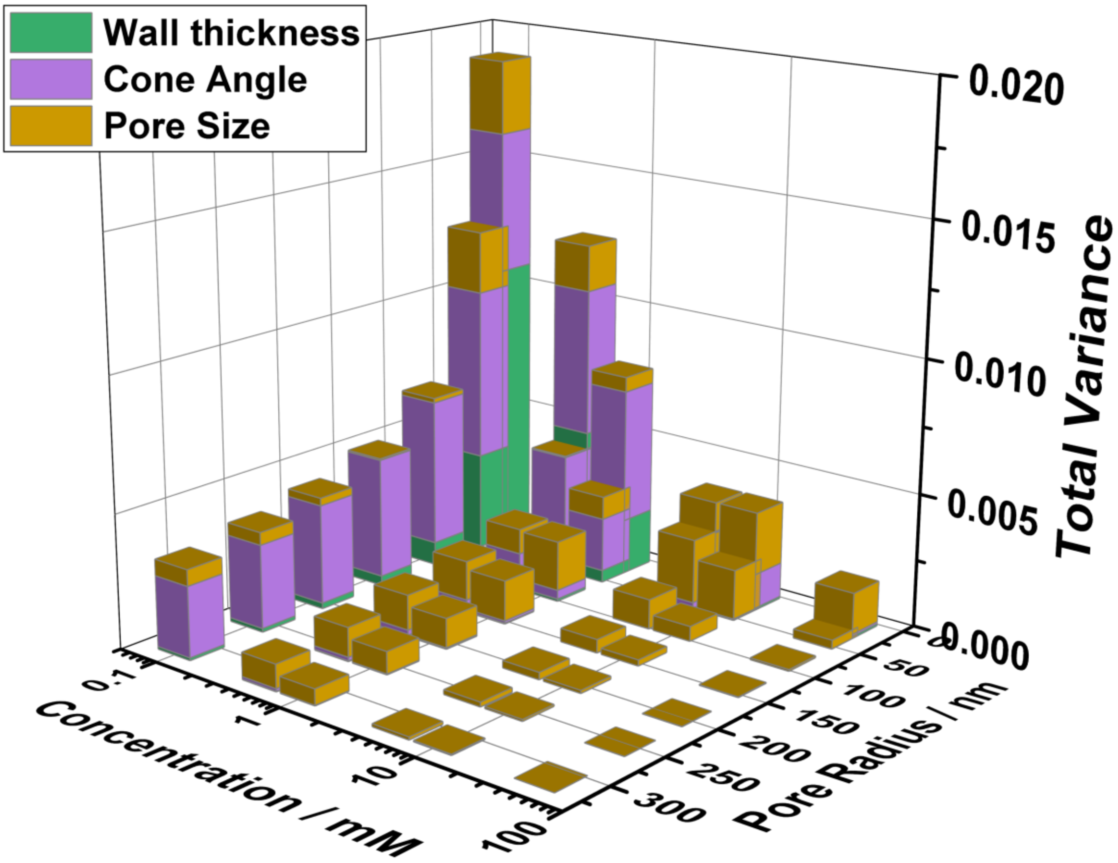

Figure S15 shows the simulated total variance, as well as the input parameters' contributions as a function of both the electrolyte concentration and pore radius.

Total Percentage Errors for Visualization of Fabrication Quality

Figure 10 in the main paper showed that once the real geometric errors associated with the fabrication of nanopore with different pore radii are considered, the 87 nm nanopores and the 232 nm nanopores showed greatest sensitivity. This is due to their fabrication quality being superior to the other studied nanopores. To illustrate this, Figure S16 give the total percentage error associated with the pore radius and cone angle for each fabrication program. The fabrication of the 87 nm pore is most reproducible, followed by that of the 232 nm pore.

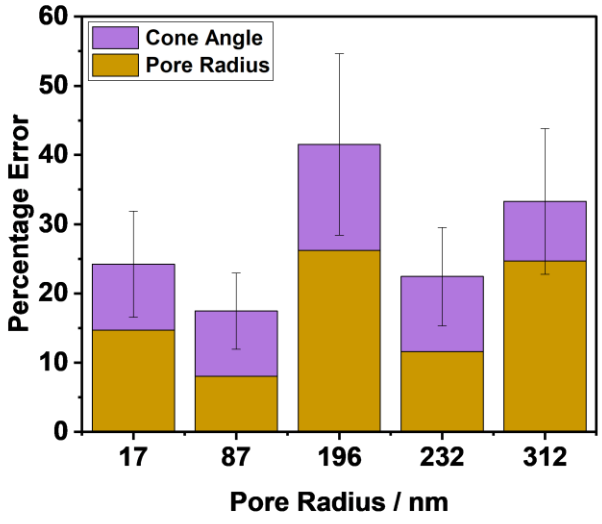

Figure S16 The total percentage associated with the fabrication originating from the variations in the pore radius and cone angle. Error bars show the confidence interval associated with the value of the percentage error, and it includes the confidence associated with the calculation of the mean and standard deviation.

Agreement of Experiments with the RR Value

In addition to the comparison of the output variance and Jeffreys Divergence values that has been presented in the main paper, here, the simulated and experimental rectification ratios are provided as the electrolyte concentration and pore radius is decreased.

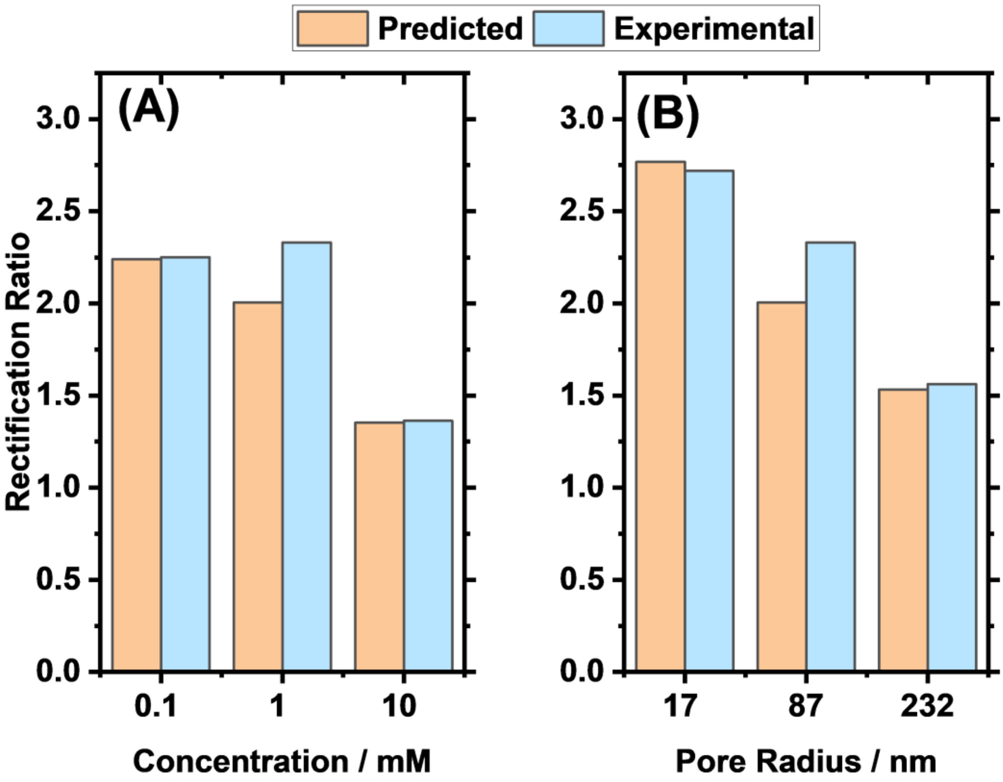

Figure S17 shows a comparison the experimental and simulated rectification ratios as a function of the electrolyte concentration and pore radius.

Experimental Distributions of the Rectification Ratio

A population (at least 60) of current-voltage curves in the presence and absence of the aminosilane analyte was recorded to obtain the distributions of the sensor output in the presence and absence of the aminosilane analyte. For this the experimental Jeffreys Divergence could be computed for comparison to the Jeffreys Divergence obtained from the model. Figure S18 shows the histograms of the experimentally obtains distributions.

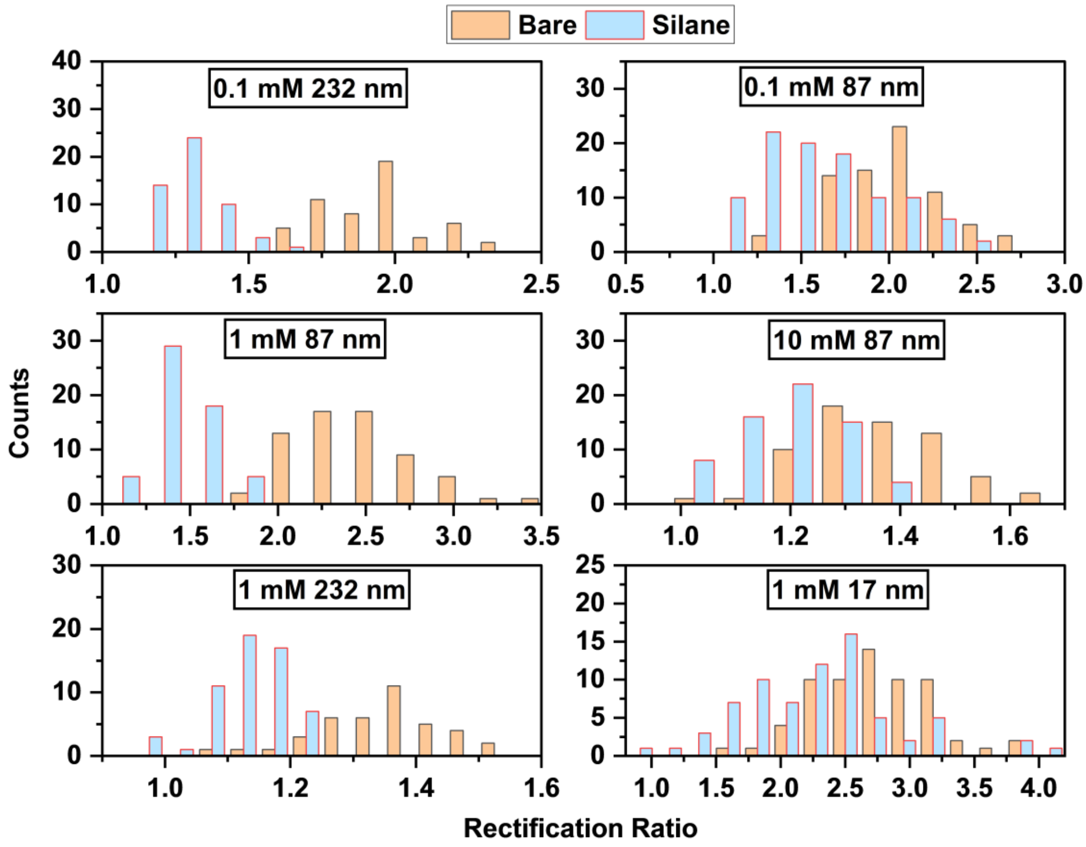

Figure S18 Histograms showing the experimentally obtained distributions of the rectification ratios for all combinations of pore radii and concentrations that were used for the experimental validation.

Experimental Jeffreys Divergence as a Function of the Applied Voltage

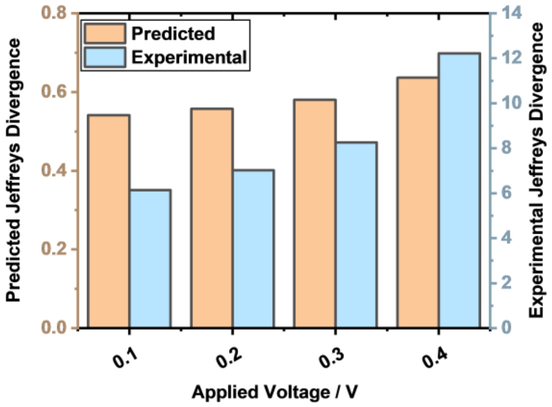

Figure S19 Comparison of experimental and simulated Jeffreys Divergence as a function of the applied voltage for the 87 nm pore at 1 mM concentration.

Recommended Operating Conditions for Different Scenarios

Figure S20 is provided as guidance for the recommended operating conditions for the ion current rectifying nanopore scenarios discussed in the main paper.

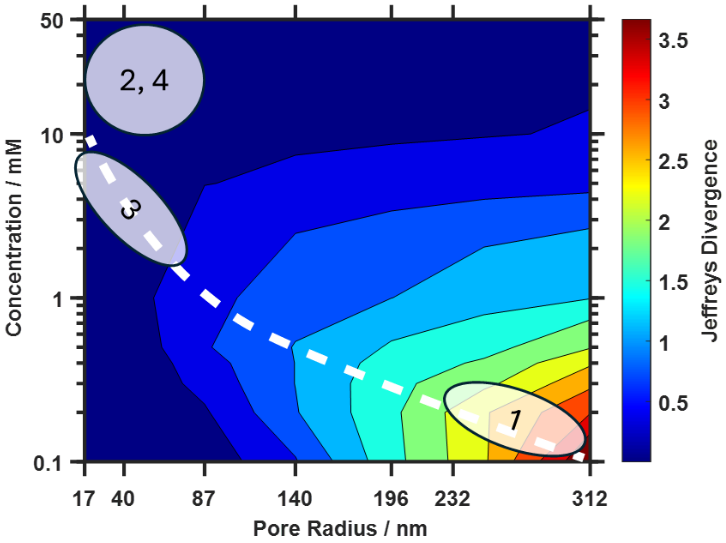

Figure S20 The recommended geometric and operating conditions for different proposed scenarios. The numbers of the highlighted regions correspond to the scenario number as discussed in the main paper.

References

(1) Duleba, D.; Johnson, R. P. Proton Enrichment and Surface Charge Dynamics in pH-Responsive Nanopipettes. *Electrochimica Acta* **2024**, *479*, 143838. https://doi.org/10.1016/j.electacta.2024.143838.

(2) Miller, D. *Estimation of Tracer Diffusion Coefficients of Ions in Aqueous Solution*; Lawrence Livermore National Lab.(LLNL), Livermore, CA (United States), 1982.

(3) Robinson, R. A.; Stokes, R. H. *Electrolyte Solutions*; Courier Corporation, 2002.

(4) Malmberg, C. G.; Maryott, A. A. Dielectric Constant of Water from 00 to 1000 C. *Journal of research of the National Bureau of Standards* **1956**, *56* (1), 1–8.

(5) Sverjensky, D. A.; Sahai, N. Theoretical Prediction of Single-Site Enthalpies of Surface Protonation for Oxides and Silicates in Water. *Geochimica et Cosmochimica Acta* **1998**, *62* (23), 3703–3716. https://doi.org/10.1016/S0016-7037(98)00262-2.

(6) Paoletti, P.; Barbucci, R.; Vacca, A. Evaluation of Substituent Effects on the Enthalpies of Protonation of Amines. An Empirical Formula of Prediction. *Journal of the Chemical Society, Dalton Transactions* **1972**, *0* (18), 2010–2013. https://doi.org/10.1039/DT9720002010.

(7) Yeh, L.-H.; Zhang, M.; Qian, S. Ion Transport in a pH-Regulated Nanopore. *Analytical chemistry*, 2013, *85*, 7527–7534.

(8) Atalay, S.; Yeh, L.-H.; Qian, S. Proton Enhancement in an Extended Nanochannel. *Langmuir*, 2014, *30*, 13116–13120.

(9) Zhang, X.; Xu, M.; Yang, J.; Hu, N. Ion Transport in pH-Regulated Double-Barreled Nanopores. *Analytical Chemistry*, 2022, *94*, 5642–5650.

(10) Iler, K. R. The Chemistry of Silica. *Solubility, polymerization, colloid and surface properties and biochemistry of silica*, 1979.

(11) Andersen, M. B.; Bruus, H.; Bardhan, J. P.; Pennathur, S. Streaming Current and Wall Dissolution over 48 h in Silica Nanochannels. *Journal of colloid and interface science*, 2011, *360*, 262–271.

(12) Wang, M.; Kang, Q.; Ben-Naim, E. Modeling of Electrokinetic Transport in Silica Nanofluidic Channels. *Analytica chimica acta*, 2010, *664*, 158–164.

(13) van der Heyden, F. H.; Stein, D.; Dekker, C. Streaming Currents in a Single Nanofluidic Channel. *Physical review letters*, 2005, *95*, 116104.

(14) Zhang, H.; He, H.-X.; Wang, J.; Mu, T.; Liu, Z.-F. Force Titration of Amino Group-Terminated Self-Assembled Monolayers Using Chemical Force Microscopy. *Appl Phys A* **1998**, *66* (1), S269–S271. https://doi.org/10.1007/s003390051143.
